# Development of infrastructure for a systemic multidisciplinary approach to study aging in retired sled dogs

**DOI:** 10.18632/aging.203600

**Published:** 2021-09-28

**Authors:** Daria I. Fleyshman, Joseph J. Wakshlag, Heather J. Huson, John P. Loftus, Natasha J. Olby, Leonid Brodsky, Andrei V. Gudkov, Ekaterina L. Andrianova

**Affiliations:** 1 Vaika, Inc., East Aurora, NY 14052, USA; 2 Cornell University College of Veterinary Medicine, Ithaca, NY 14853, USA; 3 Cornell University College of Agriculture and Life Sciences, Ithaca, NY 14853, USA; 4 North Carolina State University College of Veterinary Medicine, Raleigh, NC 27606, USA; 5 Tauber Bioinformatic Research Center, University of Haifa, Haifa, Israel; 6 Roswell Park Comprehensive Cancer Center, Buffalo, NY 14263, USA

**Keywords:** canine, senescence, frailty, longevity, healthspan

## Abstract

Canines represent a valuable model for mammalian aging studies as large animals with short lifespans, allowing longitudinal analyses within a reasonable time frame. Moreover, they develop a spectrum of aging-related diseases resembling that of humans, are exposed to similar environments, and have been reasonably well studied in terms of physiology and genetics. To overcome substantial variables that complicate studies of privately-owned household dogs, we have focused on a more uniform population composed of retired Alaskan sled dogs that shared similar lifestyles, including exposure to natural stresses, and are less prone to breed-specific biases than a pure breed population. To reduce variability even further, we have collected a population of 103 retired (8-11 years-old) sled dogs from multiple North American kennels in a specialized research facility named Vaika. Vaika dogs are maintained under standardized conditions with professional veterinary care and participate in a multidisciplinary program to assess the longitudinal dynamics of aging. The established Vaika infrastructure enables periodic gathering of quantitative data reflecting physical, physiological, immunological, neurological, and cognitive decline, as well as monitoring of aging-associated genetic and epigenetic alterations occurring in somatic cells. In addition, we assess the development of age-related diseases such as arthritis and cancer. In-depth data analysis, including artificial intelligence-based approaches, will build a comprehensive, integrated model of canine aging and potentially identify aging biomarkers that will allow use of this model for future testing of antiaging therapies.

## Canine model of mammalian aging

Medical advances during the last century have significantly extended the average lifespan and healthspan of humans. While, in large part, this has been through the development of improved treatments for specific diseases, we are also continually gaining insights into the mechanisms of aging itself. With this growing depth of knowledge, aging is no longer considered an untouchable law of nature but rather a physiological challenge that may be defeated by science and medicine. Most of our current knowledge about mammalian aging is derived from human gerontology. This provides a strong phenomenological foundation, but little insight into aging mechanisms. Mechanistic studies have been primarily conducted in the most accessible animal model, laboratory mice. Mouse models allowed the *in vitro* phenomenon of cellular senescence to be linked to systemic “inflammaging” [1, 2], led to the discovery of aging biomarkers [3, 4], allowed development of valuable experimental tools [5], and were used to demonstrate the efficacy of several antiaging approaches (i.e., mTOR inhibitors and senolytic compounds) [6–13]. However, inbred animals living in artificially healthy conditions without any environmental stresses are likely to only remotely represent naturally occurring mammalian aging [14]. Thus, extending aging research towards more relevant experimental models is desperately needed.

In this regard, the canine model provides multiple advantages. Compared to mice, the aging of dogs resembles that of humans much more closely. Like humans, aged dogs develop frailty and several age-related diseases, including obesity, chronic renal and liver pathologies, congestive heart failure, arthritis, sarcopenia, cancer, immune-mediated pathologies, and neurodegenerative diseases [15–18]. Also, like humans, dogs receive medical treatments such as surgical procedures, chemo- and radiation-therapy, dental care, and vaccinations [19, 20]. Moreover, dogs share the environment with humans and are, therefore, physiologically and psychologically closer to us than most other animal species making canines a valuable model for preclinical pharmacology. These considerations, which have been long recognized in oncology and are highlighted in the Comparative Oncology Program at the National Cancer Institute [21], are equally applicable to the study of aging. Thus, it is gratifying to see aging studies increasingly performed in canine models.

The use of canines for aging studies works towards the obvious goal of prolonging the healthspan of humans and addresses the potential for extending the healthy years of humans’ closest companions – domestic dogs. In many societies, dogs are no longer treated as mere pets but more like family members whose well-being is a significant quality-of-life (QOL) factor for their owners. The depth of such relationships is reflected in veterinary questionnaires indicating that most owners provide better medical care and comply with health check visits more rigorously for their dogs than they do for themselves [22]. Another important aspect of the role of dogs in our society is their involvement in essential human services. The extensive training required for professional assistance-, guide- and rescue-dogs (taking around 2 years) is not well balanced with their limited number of active/working years; thus, extending working dogs’ healthspans is highly desirable [23].

## Existing canine aging studies

The potential significance of canines for aging research is reflected in a growing number of studies performed in aging dog models. The largest longitudinal study to date is the Golden Retriever Lifetime Study [24], which follows a cohort of over 3,000 privately-owned Golden Retrievers from 6 months of age until their natural death. Information on the dogs’ health is collected from owners and veterinarians. Biobanking of biological samples is conducted jointly by Morris Animal Foundation, Flint Animal Cancer Center, Colorado State University, and American Humane Association (https://www.morrisanimalfoundation.org/golden-retriever-lifetime-study). The main goal of this study is to define risk factors for development of canine diseases, with a predominant focus on cancer. Started in 2015, this study has already revealed several important aspects of canine biology, such as inbreeding effects on fecundity [25] and adverse effects of gonadectomy on obesity and orthopedic complications in future life [26].

Another outstanding research platform enabling longitudinal data collection from aging dogs is the Dogs Aging Project (https://dogagingproject.org/) conducted by the University of Washington and Texas A&M University [27]. The primary goal of this study is to test in dogs the antiaging properties of rapamycin, an FDA-approved mTOR inhibitory drug that was initially developed as an immune-suppressant but showed antiaging properties in mice [5] and anti-cancer properties in mice [28–30] and humans [31]. An initial randomized, double-blind veterinary clinical trial confirmed the safety of this treatment and provided indications of potential efficacy (benefits for cardiac function) in dogs [32]. As the next step, the study is currently enrolling 500 more dogs of various genetic and environmental backgrounds to participate in a larger double-blind, placebo-controlled rapamycin study. Separately, this project plans to enroll 10,000 dogs over ten years and observe their natural aging while collecting data and samples, which will allow analysis of genetic, environmental, and veterinary factors affecting canine health and longevity.

Other studies of canine aging have tended to focus on specific pathologies rather than a systemic evaluation of the aging process. Examples include studies in the well-established field of canine cognitive dysfunction, an aging-associated neuropathological condition resembling Alzheimer’s Disease in humans [33], studies of musculoskeletal pathologies [34, 35], which are believed to be most prevalent in old canines [36], and obesity-prevention studies primarily focused on evaluating nutrition and exercise programs [37, 38]. In addition, most of these studies are cross-sectional and thus do not provide insight into the dynamics of aging-related trajectories. Therefore, additional longitudinal research broadly assessing multiple body systems is required to fill these gaps.

In planning our approach to address this need, we also wanted to overcome another limitation of most previous canine aging studies - their reliance on dogs living in private households, which adds multiple confounding factors that complicate data generation and interpretation. The diversity of environmental, psychological, and nutrition states of privately-housed dogs, as well as differences in owners’ compliance to regular check-ups and tests, introduce undesired “noise” in the data [39]. Our goal was to establish a research infrastructure free from such external variability to focus on the intrinsic factors and processes that control aging in dogs and ultimately test experimental antiaging approaches. Therefore, instead of using privately-owned and -housed canines, we chose to establish and study a population of dogs with similar prior lifestyles (retired sled dogs) housed and studied at a centralized facility with standardized optimal housing conditions and veterinary care as described further below.

## Our aging study: rationale and focus

Aging is a multi-factorial process of functional decline of various physiological systems progressing with age, which leads to a gradual decrease in resilience following stress, injury, and disease. All existing theories of aging can be divided into two categories, which explain aging primarily as either (i) an accumulation of errors and deficiencies or (ii) a genetically determined program – a “biological clock” [40]. Both categories include several hypotheses, each based on a specific mechanism. The error accumulation theory, for example, proposes that aging results from a gradual increase in damage of nuclear [41] and/or mitochondrial DNA [42] in somatic cells, an increase in levels of reactive oxygen species causing molecular and cellular damage, and increasing malfunctioning of proteasomal/lysosomal degradation resulting in accumulation of “debris” such as modified proteins [43]. These hypotheses seem to be well substantiated by everyday experience, which provides numerous examples of error accumulation in any machinery over time. However, they fail to explain the low variability in longevity among individual representatives of the same species and its insignificant extension even under the healthiest environmental and dietary conditions. This apparent contradiction supports an alternative view of aging as a genetically determined program, which is represented by several theories such as programmed impairment of regeneration capabilities due to exhaustion of somatic stem cells because of telomere reduction [44], genome destabilization by intrinsic DNA damaging, and mutation-generating mechanisms (e.g., expansion of endogenous retroelements, the importance of which has been recently demonstrated in cancer and aging [45, 46]), the activity of an epigenetic clock affecting the profile of expressed genes with age explaining a hormone production decline [47], and progressive functional decline of the immune system (immunosenescence) [48, 49].

**Figure 1:**
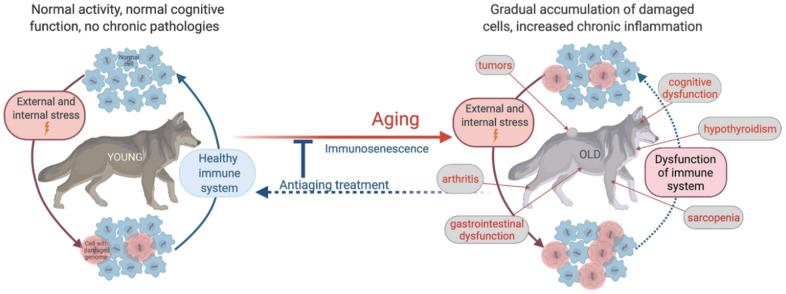
Schematic illustration of age-related physiological alterations and their underlying cellular mechanisms.

Regardless of disagreements on the mechanisms and relative impact of endogenous and exogenous factors on the rate of aging, these views are complementary, and both agree on the existence of two principal underlying aging-associated factors: progressive genome damage and immune system dysfunction. Together, these factors result in the accumulation of damaged cells followed by chronic inflammation, which leads to increased risks of infections, neoplasia, and other age-related diseases. (Figure 1). Therefore, our research strategy focuses on analyzing genome destabilization and dysfunction of the immune system occurring with age and linking them with the main universally accepted clinical signs of aging, i.e., progressive decreases in physical activity and musculoskeletal integrity, cognitive dysfunction, and development of age-related diseases.

As mentioned above, we wished to eliminate the added variability associated with studying privately housed dogs for our program. A conventional solution to this problem would be to use laboratory-bred dogs (e.g., beagles). However, this scenario still has significant drawbacks due to the animals’ narrow genetic background and lack of exposure to natural environmental pathogens leading to insufficient immune system training [14], both of which could impact critical points of natural aging processes. Using household dogs placed in a facility with unified conditions is also not ideal since they would come from highly variable prior conditions and breed backgrounds and would experience substantial emotional stress by being separated from their owners. Therefore, we chose to focus on retired working dogs raised and kept under generally similar conditions and accustomed to working with several different handlers (suggesting that their accommodation in our facility would be less stressful).

Among available working dogs, we chose sled dogs for the following reasons: (i) Genetics: Sled dogs represent a genetically distinct population that is somewhat similar to purebred breeds due to their intense selection for performance and lifestyle (outdoor housing) yet is relatively heterogeneous due to their open breeding management (dogs can and are occasionally crossbred with purebred breeds to enhance performance). This unique genetic structure provides a relatively homogeneous population to study without high levels of inbreeding confounding the assessment of aging. (ii) Environmental exposure: These dogs are raised under natural conditions allowing exposure to environmental pathogens through group interactions, thus providing an adequate model for assessing immune system aging. (iii) Adaptability: Despite originating from multiple kennels, these dogs are accustomed to generally living in a pack with less bonding to a single individual, which helps them quickly adapt to new boarding conditions. (iv) Existing background information: Due to their participation in competitions throughout their lives, there are commonly records of the physical performance of these dogs. This data will help us track any decline in their physical fitness after retirement and monitor other physiological parameters associated with this decline and whether specific treatments can restore it. Moreover, due to strong interest in the athletic features of this population, several studies have described major hematological and other physiological parameters characteristic of young sled dogs participating in racing [50, 51], making it easier to analyze changes occurring after their retirement.

## Establishment of a kennel and research facility for retired sled dogs

Development of the infrastructure to accomplish the goals described above began in 2018 with the creation of Vaika, Inc., a not-for-profit medical research entity dedicated to the aging sled dog program, and establishing a kennel and research facility on the Baker Institute campus of the College of Veterinary Medicine at Cornell University (Ithaca, NY). Under the Vaika-led program, we have enrolled and currently house/monitor 103 retired sled dogs (56 males, 47 females) ranging in age from 8 to 11 years old at the time of enrollment. This population was recruited from 23 kennels across North America and includes 57 dogs trained as sprinters, typically running <30 miles, and 47 dogs trained for distance events spanning 200-1000 miles.

Recruited dogs are housed in an 8,254-sq. ft. kennel facility that includes six 675-sq. ft. boarding rooms equipped with single-house kennels with removable dividers to facilitate co-housing of compatible animals (Supplementary Figure 1). Its veterinary suite (406 sq. ft.) includes a prep room, a surgical suite, a laboratory area, and an exam room with a wet table for dental cleanings and other non-sterile veterinary procedures (labeled as “Procedure rooms” in Supplementary Figure 1). A 464-sq. ft. room houses a gait analysis treadmill (Gait4Dogs) and a painted floor space with a ceiling-mounted camera for behavioral observations (“Playroom”). The facility has three dedicated outdoor fenced-in fields, where dogs are let out twice daily for 20-30 minutes of supervised play in groups of 3-8 compatible dogs for social enrichment and environmental exposure.

Upon arrival at the facility, we obtained owner informed consent to release the dog to Cornell University, pedigree, vaccination, and health records, and typical racing performance histories for all dogs. Dogs were acclimatized, veterinarians performed physical exams and preliminary bloodwork, females were spayed, and all dogs were placed on the same diet (Annamaet Extra 26, Annamaet Petfoods, Sellersville, PA). Vaccines and parasite treatments were updated as needed, and a synchronized protocol was established for the colony. Vaika-housed dogs are vaccinated for distemper, adenovirus, parvovirus, parainfluenza, leptospirosis (serovars *canicola*, *icterohemorrhagiae*, *grippotyphosa*, and *pomona*), and rabies virus according to American Animal Hospital Association vaccination guidelines [52]. The dogs are all fitted with flea and tick collars for external parasite control (Seresto Collar, Elanco Pharmaceuticals, Atlanta, GA) and are provided appropriate heartworm testing and preventative treatment monthly (Interceptor, Elanco Pharmaceuticals, Atlanta, GA). Routine complete blood counts and serum biochemistry testing are performed on all dogs biannually, with additional veterinary exams and treatments given as needed to maintain a proper standard of care. At the time of recruitment, dogs are scored for body condition (scale of 1-9, with 5 being ideal), and feeding adjustments are made to maintain ideal body condition and weight (measured every 3-4 weeks) since over- or under-nutrition can influence longevity.

Dogs were identified for co-housing within the indoor kennels and divided into groups for outside playtime based on their compatibility (upon recruitment, owners provided a short description of behavior peculiarities for each animal, specifying if there were signs of aggression/anxiety towards other dogs or humans). Gradual arrival of animals at the kennel facilitated the smooth introduction of new members to the colony. Any dogs displaying undesired social behavior are relocated to another kennel or playgroup. All dogs are singly housed during feeding time and co-housed as appropriate for their nature for the remainder of the day. Co-housing and playgroups continue to be updated as dogs age and circumstances change. Diseased dogs or dogs recovering after surgical procedures are isolated from regular playgroups and, whenever possible, walked individually. During severe weather conditions, outdoor playtime is substituted by indoor enrichment.

All aspects of dog maintenance, observation, testing, and treatment are performed in strict compliance with the standards of Cornell University’s Institutional Animal Care and Use Committee (Protocol #2018-0022), which follows the NIH guidelines on ethical standards for work with laboratory vertebrate animals [53]. An extensive set of standard operating procedures, including routine medical and feeding protocols for the dogs, research testing protocols, staff training protocols, and facility management protocols, have been developed for the project and have been successfully implemented during the time since Vaika’s establishment in 2018 (a description of key operating procedures are provided in Supplementary Materials).

## A collection of tests for multidimensional assessment of aging-related processes

Based on the principles of the research design described above and considering the limitations of the selected dog population, we developed a minimum list of parameters that must be assessed to achieve our goals and identified tests for the measurement of general health, physical fitness, immune system dysfunction, cognitive dysfunction, and somatic cell genome modifications. The tests and schedule of their application are presented in Table 1, with further details provided in the following sections.

**Table 1:** Schedule of assessments performed in aging sled dogs.

**Test**	**Parameters**	**Timepoints**	**Participants**
***General health***			
Physical examination	General appearance, physical palpation, weight, body condition	Every 6 months	All dogs
Complete blood counts (CBC)	Standard		
Serum biochemistry	Standard + thyroid hormones		
Morbidity	Diagnosed disease(s)	As needed	
Therapy	Treatment(s) prescribed		
Whole Body CT scans	Cancer, arthritis	Annually	
Sarcopenia assessment	Biomarkers in biopsies, CT body mass index, metabolomics		
Osteoarthritis, pain assessment	CT scan- and physical exam-based diagnostics		
***Physical fitness***			
Treadmill test	Endurance, lactate increase; heart rate resilience; cytokine response to exercising	Every 6 months	60 dogs
Pull test	Average time of pulling 1.5x bodyweight cart		
***Immune system status***			
T-cell phenotyping	% of CD4, CD8, CD25, CD28, FoxP3 cells	Annually	60 dogs
T-cell functionality by ELISPOT	IFN-y, IL-2, IL-4, IL-10, and IL-17 in response to stimulation	Annually	60 dogs
Phagocytosis by peripheral blood cells	Neutrophil and monocyte ability to phagocytose fluorescent beads	Annually	All dogs
Response to vaccination	Antibody titers following leptospirosis vaccine	Annually	All dogs
Steady state of circulating cytokines	GM-CSF, IL-2, IFN-γ, IL-6, IL-8, IL-15, IP-10, IL-10, KC-like, IL-18, MCP1, TNF-alpha	Every 6 months	60 dogs
***Cognitive dysfunction***			
Open field tests	Locomotion/activity, interaction with a person, toys and mirror	Every 9 months	All dogs
Questionnaire	Behavioral assessment by caretakers and researchers	Every 9 months	
Neuromarkers in plasma	NfL and β-amyloid variants and glial fibrillary acidic protein	Every 18 months	
Problem solving test	V-test	Every 9 months	
***Somatic cell genome modifications***			
Whole genome sequencing	Content of SINEs and LINEs	Annually	All dogs
DNA methylation	“Methylation clock” assessment		
Cellular senescence marker	*p16/Ink4a* RNA level in PBMC		

Currently, we are in the process of data collection and preliminary analysis. Analyses performed to date with the accumulating data have supported the relevance of the monitored parameters for the aging process (see below). In addition, samples from the dogs are being used to build a biobank for future research purposes. This biobank is comprised of DNA and plasma from blood and biopsies of skin, muscle, and adipose tissue collected annually throughout the animals’ lives, as well as complete tissues (both frozen and paraffin blocks), collected upon necropsy at the time of the animal’s death.

## General health

General health status is an essential indicator of aging for any mammal, including dogs and humans. This complex parameter is directly relevant to quality of life (QOL) and correlates with residual life expectancy [54, 55]. For humans, substantial effort has been put into developing indices that reduce the complex composition of general health parameters associated with aging to a single quantitative value, e.g., a frailty index [56]. Several reported approaches to establish health-defining indices for animals are based on assessing deviations of fundamental health parameters from the norm for a particular species [5, 57, 58]. However, these approaches do not work across species and do not apply to dogs, whose broad variability with respect to size, lifespan, and other characteristics makes it nearly impossible to develop an index suitable for all canines. Therefore, we had to develop a methodology appropriate for general health assessment in our aging sled dog population.

Our goal is not just to assess the state of health of a given dog but rather to dissect the aging process into its two key components: (i) declining resilience and (ii) acquisition of aging-related diseases. Aging-associated decreases in resilience manifest as impairment of an organism’s ability to return to homeostasis after intrinsic or extrinsic stresses. Reduced resilience reflects biological age but does not by itself lead to development of pathologies reducing QOL. No countermeasures targeting loss of resilience have been developed to date. The second component of aging involves pathological deficits (diseases) that are primarily the result of external influences (e.g., lifestyle, diet, infections, wounding) and are, in many cases, treatable. In aged organisms with reduced resilience, the risk of disease development increases while recovery speed diminishes; thus, QOL is progressively reduced, and the risk of death increases. Hence, the second component (disease) is typically considered the ultimate cause of death even though the first component (reduced resilience) plays such a critical role. To properly understand the aging process, it is crucial to evaluate health parameters related to each of these two aging components separately rather than combining them into a single general index.

The process of gradual loss of resilience occurring in the absence of the second component (i.e., diseases) can be considered “healthy aging.” “Healthy aging” occurs when the organism ages under optimal conditions of care, reaches its ultimate lifespan and dies from aging per se due to a complete loss of resilience. This model is described in a recent publication that explores such a scenario for humans [59]. While “healthy aging” is an extreme and rarely achievable scenario, it is essential to accurately characterize its specific parameters apart from age-related pathologies to identify biomarkers of “healthy aging” and ultimately develop antiaging therapies.

Parameters of “healthy aging” have been established for some breeds of dogs [60]. However, a comparison of reference values obtained for the two best-studied breeds, beagles and Labradors, showed substantial interbreed differences in some important (e.g., blood) parameters [60]. Thus, it is essential to establish a reference set of “healthy aging” parameters specifically for each dog model, and we see this as one of the main goals of our sled dog study. Monitoring of the second component of aging, the accumulation of deficits, is also important; this will reveal common trends of age-related diseases for our study population determined by genetic backgrounds and prior living conditions. This information can then be used to provide dogs with the proper prophylaxis and treatment of age-related pathologies (e.g., osteoarthritis) to ideally prolong their healthspans effectively and allow us to study and eventually treat the “healthy aging” component.

As shown in Table 1, we regularly assess several general health parameters in our sled dog population to characterize the onset and dynamics of clinical signs of aging. We collect data on the state of major systems in all dogs every six months, paying particular attention to pathologies known to be associated with aging in sled dogs. This data is obtained through analysis of blood samples, including assessment of red blood cell regeneration, white blood cell counts, and serum biochemistry alterations indicative of skeletal muscle integrity and renal and hepatic function. In addition, physical examinations are performed with particular focus on metabolizable energy intake and body condition, including assessment of serum thyroid hormone levels and neurologic and musculoskeletal function (sarcopenia, arthritis). Computed tomography (CT) findings related to lean body mass and arthritis are collected annually and evaluated against physical exam findings for a complete musculoskeletal and neurologic integrity assessment.

## Physical fitness

Reduced endurance is a prominent and universal sign of mammalian aging that is particularly apparent in human and animal athletes due to their loss of competitiveness. In fact, the dogs in Vaika’s population were selected based on their reduced physical performance in racing, which appeared to be due in most cases to age-related decline, or in a few cases, to musculoskeletal dysfunction. Reduced exercise tolerance in aged mammals is also reflected by an increase in the time needed to recover from physical stress. This indicates declining resilience, which, as discussed above, is the most direct aging trait. Exercise is one of the types of stress to which reduced resistance correlates with mortality in humans [61], although this has not yet been determined in dogs. Thus, assessments of physical performance and the speed of recovery from exercise stress are vital components of our aging-tracing toolset. Since our dogs are retired athletes well adapted to running, we selected treadmill and weight pull tests to measure their physical performance and tolerance to physical activity (see Table 1). Alterations in various physiological parameters in dogs after exercise have been described in earlier studies conducted in beagles [62], Labradors [63, 64], greyhounds [65], and sled dogs [66–68]. Changes in blood cell composition (erythrocytes, leukocytes, and platelets), heart rate (HR), respiratory rate, and blood levels of several cytokines, lactate, cortisol, and glucose [67, 69, 70] are defined biomarkers of physical stress responses, including the time to recover to an individual’s baseline.

In treadmill tests performed every 6 months, we measure the distance each dog covers running at its individually determined acceptable maximum speed for 20 minutes. This duration is designed to avoid endurance/exhausting conditions that would stress the animals excessively and potentially alter blood cell composition and cytokine production. We also measure HR, blood lactate and troponin levels, and the kinetics of cytokine responses to exercise. HR reflects how the cardiovascular system responds to exercise and how quickly it can return to normal. HR is measured before exercise, immediately post-exercise, and again at 2- and 20- minutes post-exercise. Increased blood lactate levels indicate muscle transition to anaerobic glycolysis and reflect the dog’s tolerance to exercise. Lactate is measured immediately pre- and post-exercise. Post-exercise troponin levels are an established marker of exercise-related cardiac injury in humans [71, 72] and are therefore used as indicators of stress placed on the cardiovascular system which is measured before and 5 minutes after the dogs’ treadmill exercise. To obtain data on cytokine responses to exercise previously documented in humans [73] and sled dogs [68, 74], we collect serum before, and 5 minutes, 2-, 4-, and 24- hours after treadmill exercise and assess levels of GM-CSF, IL-2, IFN-γ, IL-6, IL-8, IL-15, IP-10, IL-10, KC-like, IL-18, MCP1, and TNF-alpha by Immunology Multiplex Assay (MILLIPLEX MAP Canine Cytokine/Chemokine Magnetic Bead Panel on Luminex platform).

In addition to analyzing the response of the above-described physiological parameters to physical exercise, the treadmill test also allows us to study various aspects of each dog’s gait, including foot placement, pressure, and duration on the treadmill, through sensors built into the system. Gait4Dog software uses this information to generate a patented Gait4Dog Lameness Score (GLS). Treadmill sensor data and GLS scores, along with information on HR, lactate, and cytokine responses to exercise, will be used to assess aging longitudinally and cross-sectionally and analyzed in the context of other findings such as indications of osteoarthritis determined through physical exams and CT scans [75, 76].

While treadmill tests provide extensive data on physical fitness, we chose to also assess dog fitness with a simple pull test that is more similar to sled dogs’ natural work of pulling a sled. Pull tests are performed every 6 months using a simply constructed wheeled cart holding weights equal to 1.5 times the dog’s body weight. The dog wears a specially designed harness typical for sled dog weight pull competitions and is encouraged by a handler through voice commands to pull the cart for 40 ft. The dog’s average time to complete the 40 ft. pull over three successive trials is recorded. Test failures and total distances traversed are also recorded.

Our attempts to capture continuous HR, respiration, and activity measurements using multiple noninvasive activity monitors during the physical fitness tests have not yet been successful due to insufficient accuracy and reliability compared to video recordings of activity.

Before initiating the treadmill or pull test, the dogs are acclimated to the equipment, handlers, and general protocol. Between tests, dogs are conditioned/habituated every other week to each of these events for approximately 5 minutes each. This routine conditioning/habituation is intended to reduce the risk of lack of familiarity with the test conditions affecting a dog’s performance on the 6-month test date. On the other hand, routine conditioning is purposefully “low-level” (short in duration and only once every other week) to prevent any increase in the dogs’ exercise capabilities, but rather recapitulate the relatively sedentary lifestyle of most dogs and humans.

**Figure 2:**
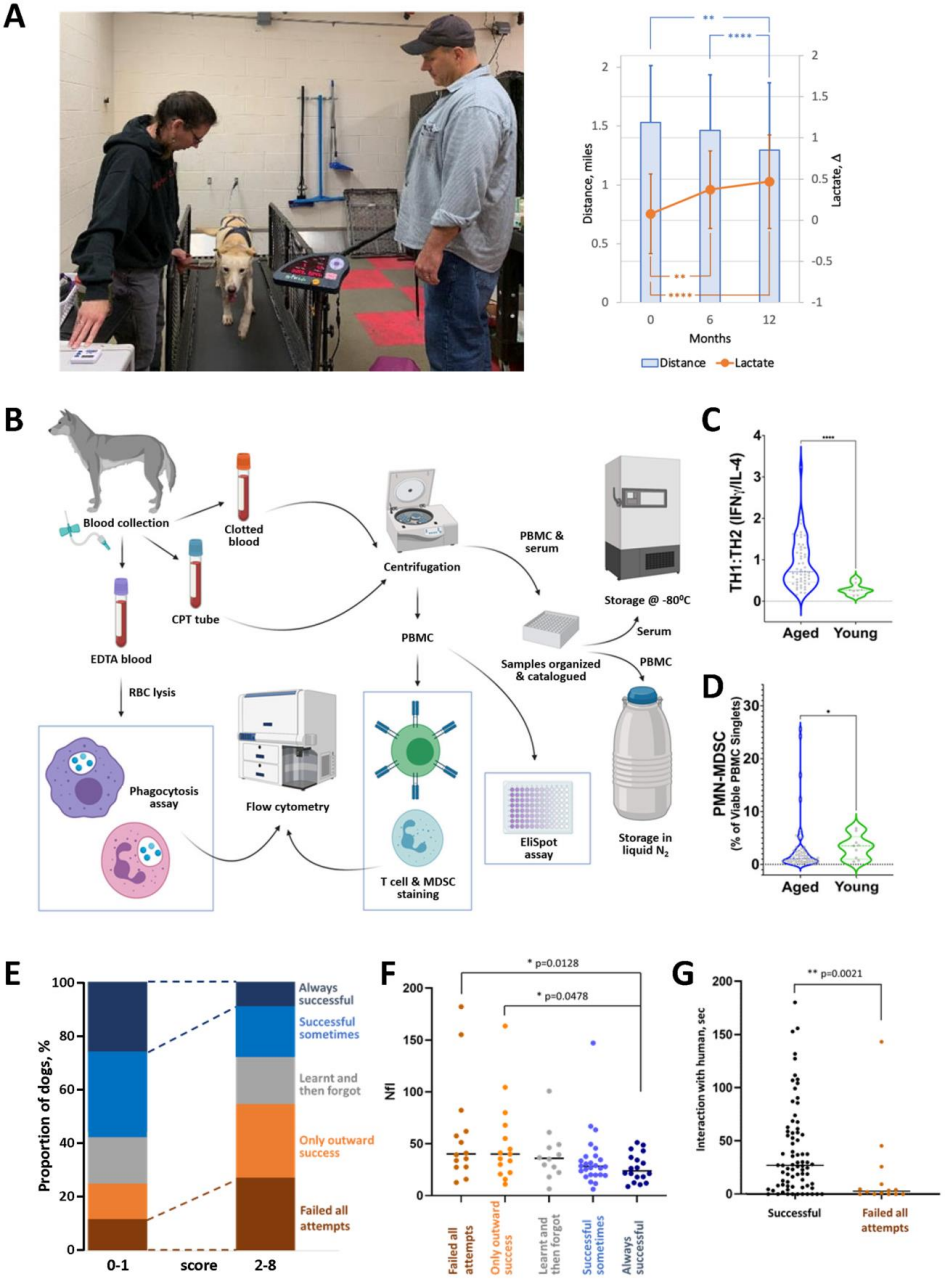
**Examples of preliminary data collected in our aging sled dog study.** (**A**) Use of the treadmill test to assess physical fitness of dogs. Left panel: example of a dog being tested on the treadmill by Drs. Huson and Wakshlag. Right panel: two independent parameters measured in the treadmill test show a gradual decline in physical performance of aged sled dogs over 12 months of observation. Mean distance (miles covered by dog running for 20 min at maximum speed adjusted for each dog) and Δ lactate (difference in blood lactate levels before and after performance) are shown. N=65-60 each timepoint. Error bars indicate standard deviation. Groups were compared by one-way ANOVA. (**B**) Overview of workflow for immunologic assays. Blood is collected from dogs into the appropriate tubes (EDTA, CPT) for corresponding downstream assays. Some assays, such as phagocytosis, T cell, MDSC staining, and EliSpot, are conducted immediately. Others, such as determination of cytokine levels and antibody titers elicited by leptospirosis vaccination, use serum and PBMC stored until analysis. (**C**) Age-dependence of T cell composition. The ratio of T cells with TH1 vs. TH2 phenotypes was determined by IFNγ/IL-4 EliSpot assays in groups of aged (8-12 years old; N=60) and young (2–4-year-old; N=10) sled dogs. Groups were compared by Mann-Whitney test. (**D**) Age-dependence of PMN-MDSC levels in peripheral blood. The percentage of PBMC expressing MDSC markers (CADO48A+CD14+) was determined by flow cytometry for the same groups of dogs as in C. Groups were compared by Mann-Whitney test. (**E**–**G**) Cognitive and neural marker tests (cross-validation of results). N=91 (**E**) Dogs’ performance in problem-solving V-tests correlates with CDS scores obtained from questionnaires completed by handlers. V-test performance is indicated by color as shown on the right y-axis; questionnaire scores reflecting the number of signs of cognitive dysfunction are shown on the x-axis. (**F**) Dogs that fail the V-test tend to have higher plasma levels of NfL (pg/ml). (**G**) Dogs that fail the V-test tend to interact less with the human in the “novel person” open field test. The “successful” group here includes dogs who were successful at least one attempt. For all panels, * - p < 0.05, **- p< 0.01, ****- p< 0.0001.

We expect that our approach to testing the physical fitness and tolerance of sled dogs to exercise will provide information on the dynamics of aging in dogs and become one of the criteria for assessing the effectiveness of antiaging therapies. Our early testing of Vaika-resident dogs supports this, which showed a statistically significant decline in physical performance in treadmill tests over 12 months (Figure 2A).

## Immune system status

The immune system protects the host organism from extrinsic (i.e., infections, wounding) and intrinsic (i.e., damaged, dead, or transformed cells) challenges. The immune system’s key role in health maintenance underscores its unique and central role in aging. The decline in immune function that develops with age termed “immunosenescence” is a fundamental component of the “healthy aging” process, defined as the loss of resilience. In the absence of adequate immune protection, what might have been a transient or asymptomatic disease in young individuals can turn into a chronic or even lethal condition [59]. Immunosenescence is a universal sign of mammalian aging, and its consequence – chronic unresolved inflammation – is an essential component of all diseases associated with aging, including atherosclerosis, osteoarthritis, diabetes type 2, cancer, and neurodegenerative and autoimmune diseases [77]. Strong systemic effects of experimental rejuvenation of the hematopoietic system illustrated the pivotal importance of declining immune function during aging [78].

Immunosenescence affects both the innate and adaptive branches of immunity. Failure of innate immunity manifests as ineffective “garbage removal” functions, resulting in the accumulation of cellular debris, elevated cancer risk, and an increased number of senescent cells [79, 80]. While immunosenescence leads to suppression of the housecleaning functions of innate immunity, it does not affect its capacity for activation; this misbalance creates conditions in which unresolved activating signals induce the development of subclinical chronic systemic inflammation, known as “inflammaging” [81].

Adaptive immunity also undergoes pronounced changes during aging. The number of naive T cells decreases, primarily due to thymic involution [82], leading to reduced diversity of the T cell receptor (TCR) repertoire. This, in turn, leads to a higher risk of infections, dampened vaccination response, and impairment of immune memory to previously encountered pathogens. At the same time, the proportion of terminally differentiated CD28- cells increases, especially within the CD8+ population [77]. The reason for this shift is likely from innate immunity dysfunction and chronic antigenic stimulation by, for example, viruses that remain latent until immunity is weakened (e.g., herpes simplex virus and cytomegalovirus in humans [83, 84]).

In general, the canine immune system undergoes similar age-related changes to that of humans [85]. However, since completed canine studies are generally less comprehensive and predominantly cross-sectional, the reliability and relative significance of various immune parameters in aging have yet to be characterized. For example, an inverted CD4/CD8 ratio is an established biomarker of high-risk mortality in aged humans, [86–88] but was not confirmed in a longitudinal study conducted in aging Labrador Retrievers [89]. In dogs, the timeline of thymus atrophy is breed- and gender-specific, and longer-lived dogs tend to maintain pools of recently emigrated thymic cells longer than short-lived dogs [90]. Therefore, immune dysfunction occurring with age may differ from breed to breed, and previously accumulated information cannot be assumed to be relevant to our sled dog population. In our aging sled dog study, we use a battery of tests to assess age-related changes in both innate and adaptive immunity longitudinally and in the context of other pathologies. These tests (see Table 1) encompass five areas of immune function and phenotype, including (i) T cell phenotype and function, (ii) humoral responses to vaccination, (iii) circulating polymorphonuclear myeloid-derived suppressor cells (PMN-MDSC), (iv) phagocytic activity of neutrophils and monocytes, and (v) serum cytokine levels. Cytokine measurements are collected every 6 months, and all other tests are performed annually. The workflow for collection and processing of blood samples for all immunologic assays is shown in Figure 2B.

We assess T cell phenotype by flow cytometry after staining for the following markers: CD3, CD4, CD8, CD25, CD28, and FoxP3. These markers define major T lymphocyte populations, including T helper cells (CD4^+^), cytotoxic T cells (CD8^+^), and T regulatory cells (CD4^+^, CD25^+^, FoxP3^+^). We use EliSpot assays to evaluate T cell function by measuring proliferation of five cytokine-secreting cell types (IL-2, IL-4, IL-10, IL-17, or IFNγ) in response to the mitogens concanavalin-A or phytohemagglutinin. Sled dogs’ humoral responses to vaccination will be determined by measuring specific antibody titers in serum samples collected just before and one month after annual vaccination with a four-serovar leptospirosis vaccine. Circulating PMN-MDSC are increased in the blood of dogs with tumors, a significant age-related category of canine disease [91, 92]. We measure the presence of these cells by staining the PBMC fraction of density centrifugation-separated cells with a canine neutrophil-specific monoclonal antibody (clone CADO48A) followed by flow cytometry. Phagocytic activity, primarily executed by neutrophils, is an essential component of host responses against microorganisms [93, 94]. We measure this effector function in PBMC samples using an assay in which Fc receptor-mediated phagocytosis of immunoglobulin-coated fluorescent latex beads is quantified by flow cytometry. Finally, analysis of serum cytokine concentrations provides an overview of the systemic immunologic milieu, including inflammation. This data is obtained using frozen serum samples and the Milliplex Canine Cytokine/Chemokine panel, which simultaneously measures 13 canine cytokines and chemokines: GM-CSF, IFN-γ, IL-2, IL-6, IL-7, IL-8, IL-10, IL-15, IL-18, IP-10, KC-like, MCP-1, and TNFα.

Our preliminary analysis of data from these assays demonstrated statistically significant age-dependent changes in immune parameters, including T cell phenotype (Figure 2C) and presence of PMN-MSDC (Figure 2D) between populations of young sled dogs (2-4 years old) and aged sled dogs (8-12 years old). We expect a complete analysis of immunologic data collected longitudinally over a multi-year timeframe will provide a robust picture of the dynamics of age-related immune decline in sled dogs.

## Cognitive tests

Cognitive decline is considered a major age-related disease; therefore, monitoring the dynamics of this pathology in our sled dog population is an essential aspect of this study. Canine cognitive dysfunction syndrome (CDS) is a neuropathological condition that progresses with age and affects a dog’s health and QOL [33]. It is diagnosed in up to 60% of aged canines, with no gender or breed differences and 11 years of age being the most common onset of owner-detected behavior changes [95, 96]. CDS is manifested by behavior and personality changes summarized by the acronym DISHA: disorientation; changes in interactions with owners, other pets, or the environment; sleep-wake cycle disturbances; house-soiling, and changes in activity [97, 98]. While CDS is usually diagnosed based on owner-filled questionnaires on DISHA parameters [97], other measures are also available. These include tests of spontaneous activity [99–101], novelty seeking [102], social interactions [99, 103], and problem solving [104], which have been developed to more objectively measure behavior patterns associated with CDS. In addition, brain atrophy and neuron loss accompanying CDS are characterized by deposition of β-amyloid (Aβ) plaques [103, 105–108] and release of the light chain of neurofilament (Nfl), both of which can be detected in circulation [103, 109, 110].

Our study cannot entirely rely on behavioral and QOL questionnaires since we do not have detailed information regarding individual dogs’ habits and behavior in the past and cannot distinguish between pre-existing and acquired conditions. However, we can detect age-related changes occurring during the study and classify dogs into behavioral categories based on questionnaires completed by our staff dog handlers (Supplementary Figure 2). Importantly, repeated replies within reports provided by different handlers add statistical value to the study. The findings from questionnaires will be supplemented by additional, more objective tests related to CDS, including open field tests, detection of neurodegenerative biomarkers in plasma, and problem-solving “V” tests (see Table 1).

Several studies have shown that locomotor activity is affected by aging and cognitive dysfunction [99–101]]: while elder dogs tend to decrease overall locomotor activity, those with cognitive decline increase locomotion and their movements are characterized as aimless and not affected by the introduction of stimuli. Open field tests developed by Rosado et al. assess these aspects of locomotion by monitoring spontaneous activity of dogs in different environments [111, 112]. This testing platform was selected for use in our study since sled dogs differ from typical pet dogs in their upbringing (living outside of an owner’s house, not used to playing with toys, not trained for typical pet dog commands, but responding to mushing-related tasks) and in being less consistently food-motivated. Therefore, we could not utilize some widely accepted behavior testing approaches, such as tests involving owner-pet interaction or learning new commands, and instead focused on tests that did not require specific or repeated training.

We perform open field tests every nine months, with each dog participating in four consecutive tests in (i) an empty room, (ii) a room with toys, (iii) a room with a mirror, and (iv) a room with a novel person that is not allowed to interact with the dog (see Supplementary Materials for detailed information). The testing room floor is divided into equal squares, enabling quantification of the dog’s locomotion by calculating the number of squares crossed during a given amount of time. Patterns of locomotion (time spent sitting/standing/laying down vs. walking/running) are also recorded. Importantly, changes in locomotor activity might not necessarily reflect cognitive decline. For example, decreased activity might be due to sickness. Therefore, in our analysis, we incorporate physical exam results and information on the onset of novel health issues (e.g., arthritis and related lameness or vision decline) and blood parameters that might affect the dogs’ mood and well-being (e.g., thyroid gland function). Other parameters tracked in open field tests include time spent by the dog at defined parts of the room (door zone, corners, central area) with or without additional stimuli (mirror, toys, or novel human) and the extent of the dog’s interaction with mirror, toys, or human. The latter information allows us to test novelty seeking, which was shown to decline with age [102], and changes in social behavior (decreased interest in petting, avoiding contacts, or lack of greeting behavior), which serves as the most prominent factor in CDS diagnosis [99, 103].

Open field tests provide a comprehensive source of information, which after digitization, can be used for (i) classifying dogs into behavioral categories and (ii) assessing changes in dogs’ behavior with time. Such dynamics and other tests run in parallel will allow us to reveal specific behavioral patterns detectable in open field tests that most reliably reflect age-related cognitive decline.

We also test our dogs using a spatial problem-solving detour task (“V-test”) in which a treat is separated from the dog by a transparent V-shaped fence such that the animal can see but not directly access the treat. To reach the treat, the dog needs to move around the barrier. Our test conditions are classified as “continuously visible goal locomotor detours” meaning that the goal (treat) is visible all the time, and the dog needs to move in a counterintuitive direction to get it. Tests of this type assess inhibitory control (ability to decide against walking to the goal in a straight line), route planning, and learning [104].

We chose to use the V-test since it does not require additional training and enables clear quantitative interpretations. It was previously used with populations of dingoes [113] and appears to be suitable for testing dogs that are less human-oriented than household dogs. A detailed description of our version of the V-test is provided in Supplementary Materials. Our preliminary analysis shows that dogs’ performance in the V-test correlates well with independent assessment methods of their cognitive states, such as questionnaires completed by care providers (Figure 2E) and measurements of interaction with a novel human in open field tests (Figure 2G).

Finally, we measure two plasma biomarkers relevant to pathological processes accompanying CDS in addition to questionnaires and behavior-based testing. Characteristics of CDS include brain atrophy and neuron loss [114], deposition of β-amyloid (Aβ) plaques [105] with the density and tissue distribution of plaques correlating with disease severity [103, 115], increased local inflammation, and microglia activation [108], and occasionally neurofibrillary tangles formed by phosphorylated tau aggregates [108]. As in humans, Aβ deposits accumulate in the canine brain with aging [106], and this accumulation, as well as the density and load of amyloid, correlates with the severity of cognitive decline [103, 105, 108]. The ratio of the larger Aβ42 form to more abundant Aβ40 form in cerebrospinal fluid (CSF) is a good predictor of Aβ load in the brain [115]. Several studies assessed if plasma circulating Aβ might serve as a CDS biomarker and confirmed that, as in humans, plasma Aβ levels were higher in dogs with healthy aging and lower in cognitively impaired animals [110, 116]. These observations suggest that the relationship between amyloid precursor protein (APP) and cognitive dysfunction is likely similar in canines and humans [reviewed in [117] and [118].

Another biomarker of cognitive decline currently being evaluated in humans [119, 120] and dogs [103, 109] is the light chain of neurofilament (NfL), a neuronal cytoskeletal protein that is integral to neuronal structure and function. As a protein released upon neuronal damage into systemic circulation, NfL is considered a potential biomarker of neurodegeneration. Work by Panek et al. [109] demonstrated a gradual increase in plasma NfL levels over the course of healthy canine aging, with a significant increase in cognitively impaired dogs.

Plasma concentrations of Aβ and NfL are measured every 18 months in all dogs in Vaika’s population using a previously described protocol [109]. While our goal is to collect longitudinal data on these two biomarkers, preliminary data obtained at a single timepoint across our population confirmed previously reported age-related differences in both parameters. This suggests that the markers will be helpful for further longitudinal study in our cohort. We also observed correlation of NfL levels with other parameters assessed, providing cross-validation of tests (Figure 2F).

These biomarkers are considered complementary to functional cognition testing and will be integrated within a coordinated toolset enabling cross-validation of results and defining the relative value of individual assays. Initial assessment of correlations between questionnaire responses and V-test results revealed the value of this type of pooled analysis (Figure 2E), as similar conclusions emerged from unrelated approaches.

Importantly, since dogs participate in the study until the time of their natural death, we will be able to collect brain tissue at the time of necropsy and examine it for the presence of amyloid and signs of inflammation (amyloid deposition has been shown to lead to microglial activation and local inflammation [103]). This will allow us to further confirm the reliability of tested biomarkers and the scoring of behavior tests.

Together, the results of these tests will provide a robust and comprehensive characterization of age-related cognitive decline in sled dogs. This will enable analysis of connections between cognitive decline and other aspects of aging evaluated in the study and potentially be useful in future testing of the efficacy of antiaging and CDS-preventive treatments.

## Somatic cell genome modifications

Aging of mammals is associated with the gradual accumulation of cells with genetic and epigenetic changes. A progressive increase in point mutations during aging was recently directly demonstrated by genomic analysis of DNA from somatic cells of humans [121]. Specific changes in DNA methylation patterns have also been consistently observed during aging of humans and mice, leading to the concept of a “methylation clock” [122] that has been validated in both species [123–125]. Cells with damaged DNA and altered epigenetic control undergo a phenotypic transition known as cellular senescence [126, 127], which is accompanied by constitutive activation of NF-κB- and interferon-driven transcriptional responses [45], resulting in secretion of pro-inflammatory factors and establishment of a so-called SASP (senescence-associated secretory phenotype) [128]. These well-documented phenomena provide a mechanistic foundation integrating multiple aspects of mammalian aging [129]. Characterization of these processes in aging sled dogs is important for putting this model in the context of other aging studies, finding explanations for the shorter longevity of dogs vs. other species of similar body size [130, 131], and identifying biomarkers of somatic cell alterations that could be used for correlative analysis vis-à-vis other manifestations of aging. To reach these goals, we will analyze peripheral blood samples collected annually from sled dogs using three classes of research tools: (i) quantitation of mutagenic and inflammaging-inducing retrotransposition activity of SINE and LINE elements, (ii) assessment of age-related changes in DNA methylation (“methylation clock”), and (iii) measurement of *p16/Ink4a* gene expression, a biomarker of cellular senescence.

**Figure 3:**
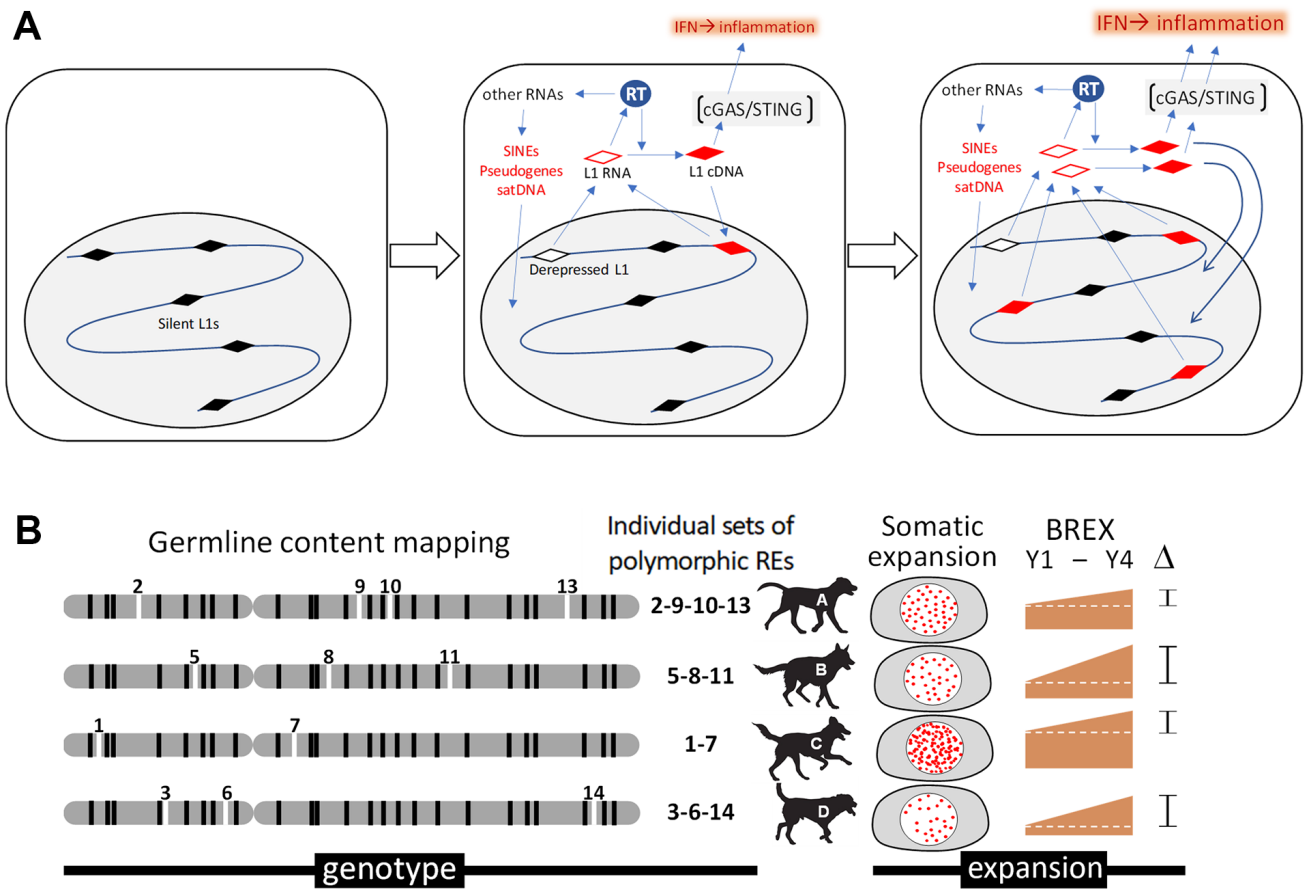
**Analysis of retrotransposons in somatic cells of aging dogs.** (**A**) Schematic depiction of a hypothetical connection between retrotransposon expansion and inflammaging. Activity of the reverse transcriptase (RT) encoded by LINE-1 (L1) retrotransposons results in insertional mutagenesis and cGAS-STING-mediated interferon type I (IFN) response and inflammation. (**B**) Illustration of our approach assessing the association between retrotransposon content and aging. Mosaicism in retrotransposon content among blood cells during aging will be assessed through computational bioinformatic analysis of data from whole genome sequencing of DNA isolated from the same dog at 2-year intervals. Determination of germline SINE and LINE-1 contents for individual dogs (shown as “genotype” for individual dogs, A-D) will be followed by quantitation of novel somatic copies of retrotransposons (new insertions indicated as “somatic expansion”). This will allow us to establish a Blood Retrobiome Expansion index (BREX) reflecting the number of somatic integrations in each DNA sample that can be tracked over time (e.g., ΔBREX between years 1 and 4 (Y1-Y4) in the figure) and compared with biological age assessment approaches based on DNA methylation and senescence marker analyses.

Our analysis of retrotransposition is based on studies of mechanisms underlying genomic alterations of somatic cells that revealed a significant role of endogenous retrotransposons, especially the LINE-1 family of repeats, as intrinsic mutagens [132, 133]. Rare spontaneous activation of LINE-1 expression, normally suppressed at an epigenetic level, drives retrotransposition of not only LINE-1 elements themselves but also the SINE family of retroelements, pericentromeric satellite DNA, and processed pseudogenes (intronless, promoterless copies of mRNAs) [134]. Together, these sequences, referred to as the “retrobiome,” comprise nearly half of mammalian DNA. In addition to causing genomic instability through the insertion of new element copies into the genome, retrobiome activation induces an inflammatory response via cGAS-STING-mediated induction of interferon signaling, which contributes to “inflammaging” [45, 46] (Figure 3A). Together, these findings led us to hypothesize that the number of SINE and LINE-1 element copies in a genome increases in an age-related manner and may be considered a “somatic genetic aging clock.” To test this hypothesis, we have developed a computational bioinformatic toolset that allows us to analyze the composition and dynamics of SINE and LINE-1 elements in dog genomes based on whole genome sequencing data. This approach is illustrated in Figure 3B.

As described above, the paradigm of a methylation clock is well established in humans and mice. In canines, this has only been looked at in relatively small cross-sectional datasets; however, findings of principal similarities in the genomic distribution of changes in DNA methylation between canines (dogs and wolves) and humans or mice suggests that the methylation clock works similarly across mammalian species [135, 136]. To confirm this and compare methylation clock activity with other markers and signs of canine aging, we will analyze age-related alterations in DNA methylation in PBMC collected annually from our population of retired sled dogs using the methods described in our previous work [46].

Finally, we will evaluate biomarkers of senescent cells since their accumulation in somatic tissues has been linked to mammalian aging [79, 137, 138]. Senescent cells undergo epigenetic transitions detected as modifications of chromatin structure that result in complex transcriptional profile changes. Activation of *p16/Ink4a* gene expression is a universally accepted and easily measurable indicator of cellular senescence reflecting epigenetic alterations associated with this cell aging phenotype [139]. We will measure p16/Ink4a RNA levels in PBMC longitudinally collected from aging dogs using quantitative RT-PCR and deep RNA sequencing methods. The latter approach will allow us to analyze p16/Ink4a expression in the context of other age-related changes in gene expression.

These data will allow us to look for coordination among genetic, epigenetic, and transcriptional age-related alterations and identify potential correlations between the “methylation clock,” the “somatic genetic aging clock,” and immunosenescence as well as parameters evaluated in other branches of the study (e.g., inflammation, cognitive decline, neurological aging markers, physical frailty).

## CONCLUSIONS

Our ongoing program will generate a large volume of data covering, in a longitudinal fashion, multiple parameters related to the general health, physical fitness, immune status, cognitive function, and somatic cell genetic and epigenetic changes of 103 Vaika-resident retired sled dogs. All the obtained data will be put in a digitized format into a sled dog aging database (SDAD), which will become the source for integrative analyses aimed at putting the totality of accumulated information into the context of major mechanistic concepts of aging (e.g., the role of senescent cells, activity of endogenous retrotransposons, immunosenescence, neurodegeneration). We expect that these analyses will allow us to (i) characterize the mechanism(s) and regulation of canine aging, (ii) identify parameters and biomarkers suitable for assessment of biological age, and (iii) define factors that may act as aging accelerators or decelerators. We plan, as a result, to create a biomarker-based methodology for objectively assessing biological age in our canine population. This should allow robust estimation of the efficacy of antiaging treatments in future trials based on biomarker responses without waiting for animals to reach their ultimate lifespan. Overall, the infrastructure established for this study provides an important advance in the use of canines, an ideal animal model for aging research that can be translated to human health.

## Supplementary Material

Supplementary Material

## References

[1] Burd CE, Sorrentino JA, Clark KS, Darr DB, Krishnamurthy J, Deal AM, Bardeesy N, Castrillon DH, Beach DH, Sharpless NE. Monitoring tumorigenesis and senescence *in vivo* with a p16(INK4a)-luciferase model. Cell. 2013; 152:340–51. 10.1016/j.cell.2012.12.010 23332765 PMC3718011

[2] Hall BM, Balan V, Gleiberman AS, Strom E, Krasnov P, Virtuoso LP, Rydkina E, Vujcic S, Balan K, Gitlin I, Leonova K, Polinsky A, Chernova OB, Gudkov AV. Aging of mice is associated with p16(Ink4a)- and β-galactosidase-positive macrophage accumulation that can be induced in young mice by senescent cells. Aging (Albany NY). 2016; 8:1294–315. 10.18632/aging.100991 27391570 PMC4993332

[3] Frescas D, Roux CM, Aygun-Sunar S, Gleiberman AS, Krasnov P, Kurnasov OV, Strom E, Virtuoso LP, Wrobel M, Osterman AL, Antoch MP, Mett V, Chernova OB, Gudkov AV. Senescent cells expose and secrete an oxidized form of membrane-bound vimentin as revealed by a natural polyreactive antibody. Proc Natl Acad Sci USA. 2017; 114:E1668–77. 10.1073/pnas.1614661114 28193858 PMC5338544

[4] Hall BM, Gleiberman AS, Strom E, Krasnov PA, Frescas D, Vujcic S, Leontieva OV, Antoch MP, Kogan V, Koman IE, Zhu Y, Tchkonia T, Kirkland JL, et al. Immune checkpoint protein VSIG4 as a biomarker of aging in murine adipose tissue. Aging Cell. 2020; 19:e13219. 10.1111/acel.13219 32856419 PMC7576241

[5] Antoch MP, Wrobel M, Kuropatwinski KK, Gitlin I, Leonova KI, Toshkov I, Gleiberman AS, Hutson AD, Chernova OB, Gudkov AV. Physiological frailty index (PFI): quantitative in-life estimate of individual biological age in mice. Aging (Albany NY). 2017; 9:615–26. 10.18632/aging.101206 28325885 PMC5391222

[6] Demaria M, Ohtani N, Youssef SA, Rodier F, Toussaint W, Mitchell JR, Laberge RM, Vijg J, Van Steeg H, Dollé ME, Hoeijmakers JH, de Bruin A, Hara E, Campisi J. An essential role for senescent cells in optimal wound healing through secretion of PDGF-AA. Dev Cell. 2014; 31:722–33. 10.1016/j.devcel.2014.11.012 25499914 PMC4349629

[7] Baker DJ, Wijshake T, Tchkonia T, LeBrasseur NK, Childs BG, van de Sluis B, Kirkland JL, van Deursen JM. Clearance of p16Ink4a-positive senescent cells delays ageing-associated disorders. Nature. 2011; 479:232–36. 10.1038/nature10600 22048312 PMC3468323

[8] Baker DJ, Childs BG, Durik M, Wijers ME, Sieben CJ, Zhong J, Saltness RA, Jeganathan KB, Verzosa GC, Pezeshki A, Khazaie K, Miller JD, van Deursen JM. Naturally occurring p16(Ink4a)-positive cells shorten healthy lifespan. Nature. 2016; 530:184–89. 10.1038/nature16932 26840489 PMC4845101

[9] Farr JN, Xu M, Weivoda MM, Monroe DG, Fraser DG, Onken JL, Negley BA, Sfeir JG, Ogrodnik MB, Hachfeld CM, LeBrasseur NK, Drake MT, Pignolo RJ, et al. Targeting cellular senescence prevents age-related bone loss in mice. Nat Med. 2017; 23:1072–79. 10.1038/nm.4385 28825716 PMC5657592

[10] Schafer MJ, White TA, Iijima K, Haak AJ, Ligresti G, Atkinson EJ, Oberg AL, Birch J, Salmonowicz H, Zhu Y, Mazula DL, Brooks RW, Fuhrmann-Stroissnigg H, et al. Cellular senescence mediates fibrotic pulmonary disease. Nat Commun. 2017; 8:14532. 10.1038/ncomms14532 28230051 PMC5331226

[11] Childs BG, Baker DJ, Wijshake T, Conover CA, Campisi J, van Deursen JM. Senescent intimal foam cells are deleterious at all stages of atherosclerosis. Science. 2016; 354:472–77. 10.1126/science.aaf6659 27789842 PMC5112585

[12] Bussian TJ, Aziz A, Meyer CF, Swenson BL, van Deursen JM, Baker DJ. Clearance of senescent glial cells prevents tau-dependent pathology and cognitive decline. Nature. 2018; 562:578–82. 10.1038/s41586-018-0543-y 30232451 PMC6206507

[13] Harrison DE, Strong R, Sharp ZD, Nelson JF, Astle CM, Flurkey K, Nadon NL, Wilkinson JE, Frenkel K, Carter CS, Pahor M, Javors MA, Fernandez E, Miller RA. Rapamycin fed late in life extends lifespan in genetically heterogeneous mice. Nature. 2009; 460:392–95. 10.1038/nature08221 19587680 PMC2786175

[14] Abolins S, King EC, Lazarou L, Weldon L, Hughes L, Drescher P, Raynes JG, Hafalla JC, Viney ME, Riley EM. The comparative immunology of wild and laboratory mice, Mus musculus domesticus. Nat Commun. 2017; 8:14811. 10.1038/ncomms14811 28466840 PMC5418598

[15] Fleming JM, Creevy KE, Promislow DE. Mortality in north american dogs from 1984 to 2004: an investigation into age-, size-, and breed-related causes of death. J Vet Intern Med. 2011; 25:187–98. 10.1111/j.1939-1676.2011.0695.x 21352376

[16] Hoffman JM, Creevy KE, Franks A, O’Neill DG, Promislow DE. The companion dog as a model for human aging and mortality. Aging Cell. 2018; 17:e12737. 10.1111/acel.12737 29457329 PMC5946068

[17] Gilmore KM, Greer KA. Why is the dog an ideal model for aging research? Exp Gerontol. 2015; 71:14–20. 10.1016/j.exger.2015.08.008 26325590

[18] Sándor S, Kubinyi E. Genetic Pathways of Aging and Their Relevance in the Dog as a Natural Model of Human Aging. Front Genet. 2019; 10:948. 10.3389/fgene.2019.00948 31681409 PMC6813227

[19] Withrow SJ, Wilkins RM. Cross talk from pets to people: translational osteosarcoma treatments. ILAR J. 2010; 51:208–13. 10.1093/ilar.51.3.208 21131721

[20] Rankin KS, Starkey M, Lunec J, Gerrand CH, Murphy S, Biswas S. Of dogs and men: comparative biology as a tool for the discovery of novel biomarkers and drug development targets in osteosarcoma. Pediatr Blood Cancer. 2012; 58:327–33. 10.1002/pbc.23341 21990244

[21] Dewhirst MW, Page RL. Editorial: Emerging Translational Opportunities in Comparative Oncology with Companion Canine Cancers. Front Oncol. 2020; 10:270. 10.3389/fonc.2020.00270 32185136 PMC7058795

[22] Janke N, Coe JB, Bernardo TM, Dewey CE, Stone EA. Pet owners’ and veterinarians’ perceptions of information exchange and clinical decision-making in companion animal practice. PLoS One. 2021; 16:e0245632. 10.1371/journal.pone.0245632 33524061 PMC7850489

[23] Creevy KE, Austad SN, Hoffman JM, O’Neill DG, Promislow DE. The Companion Dog as a Model for the Longevity Dividend. Cold Spring Harb Perspect Med. 2016; 6:a026633. 10.1101/cshperspect.a026633 26729759 PMC4691800

[24] Guy MK, Page RL, Jensen WA, Olson PN, Haworth JD, Searfoss EE, Brown DE. The Golden Retriever Lifetime Study: establishing an observational cohort study with translational relevance for human health. Philos Trans R Soc Lond B Biol Sci. 2015; 370:20140230. 10.1098/rstb.2014.0230 26056371 PMC4581032

[25] Chu ET, Simpson MJ, Diehl K, Page RL, Sams AJ, Boyko AR. Inbreeding depression causes reduced fecundity in Golden Retrievers. Mamm Genome. 2019; 30:166–72. 10.1007/s00335-019-09805-4 31115595 PMC6606663

[26] Simpson M, Albright S, Wolfe B, Searfoss E, Street K, Diehl K, Page R. Age at gonadectomy and risk of overweight/obesity and orthopedic injury in a cohort of Golden Retrievers. PLoS One. 2019; 14:e0209131. 10.1371/journal.pone.0209131 31314808 PMC6636707

[27] Lee MB, Kaeberlein M. Translational Geroscience: From invertebrate models to companion animal and human interventions. Transl Med Aging. 2018; 2:15–29. 10.1016/j.tma.2018.08.002 32368707 PMC7198054

[28] Comas M, Toshkov I, Kuropatwinski KK, Chernova OB, Polinsky A, Blagosklonny MV, Gudkov AV, Antoch MP. New nanoformulation of rapamycin Rapatar extends lifespan in homozygous p53-/- mice by delaying carcinogenesis. Aging (Albany NY). 2012; 4:715–22. 10.18632/aging.100496 23117593 PMC3517942

[29] Antoch MP, Wrobel M, Gillard B, Kuropatwinski KK, Toshkov I, Gleiberman AS, Karasik E, Moser MT, Foster BA, Andrianova EL, Chernova OV, Gudkov AV. Superior cancer preventive efficacy of low versus high dose of mTOR inhibitor in a mouse model of prostate cancer. Oncotarget. 2020; 11:1373–87. 10.18632/oncotarget.27550 32341756 PMC7170500

[30] Komarova EA, Antoch MP, Novototskaya LR, Chernova OB, Paszkiewicz G, Leontieva OV, Blagosklonny MV, Gudkov AV. Rapamycin extends lifespan and delays tumorigenesis in heterozygous p53+/- mice. Aging (Albany NY). 2012; 4:709–14. 10.18632/aging.100498 23123616 PMC3517941

[31] Xie J, Wang X, Proud CG. mTOR inhibitors in cancer therapy. F1000Res. 2016; 5:F1000. 10.12688/f1000research.9207.1 27635236 PMC5007757

[32] Urfer SR, Kaeberlein TL, Mailheau S, Bergman PJ, Creevy KE, Promislow DE, Kaeberlein M. A randomized controlled trial to establish effects of short-term rapamycin treatment in 24 middle-aged companion dogs. Geroscience. 2017; 39:117–27. 10.1007/s11357-017-9972-z 28374166 PMC5411365

[33] Landsberg GM, Nichol J, Araujo JA. Cognitive dysfunction syndrome: a disease of canine and feline brain aging. Vet Clin North Am Small Anim Pract. 2012; 42:749–68. 10.1016/j.cvsm.2012.04.003 22720812

[34] Pagano TB, Wojcik S, Costagliola A, De Biase D, Iovino S, Iovane V, Russo V, Papparella S, Paciello O. Age related skeletal muscle atrophy and upregulation of autophagy in dogs. Vet J. 2015; 206:54–60. 10.1016/j.tvjl.2015.07.005 26257260

[35] Frye CW, Shmalberg JW, Wakshlag JJ. Obesity, Exercise and Orthopedic Disease. Vet Clin North Am Small Anim Pract. 2016; 46:831–41. 10.1016/j.cvsm.2016.04.006 27289253

[36] Caron-Lormier G, England GC, Green MJ, Asher L. Using the incidence and impact of health conditions in guide dogs to investigate healthy ageing in working dogs. Vet J. 2016; 207:124–30. 10.1016/j.tvjl.2015.10.046 26616425

[37] Phungviwatnikul T, Valentine H, de Godoy MR, Swanson KS. Effects of diet on body weight, body composition, metabolic status, and physical activity levels of adult female dogs after spay surgery. J Anim Sci. 2020; 98:skaa057. 10.1093/jas/skaa057 32064516 PMC7070154

[38] Freeman LM, Abood SK, Fascetti AJ, Fleeman LM, Michel KE, Laflamme DP, Bauer C, Kemp BL, Van Doren JR, Willoughby KN. Disease prevalence among dogs and cats in the United States and Australia and proportions of dogs and cats that receive therapeutic diets or dietary supplements. J Am Vet Med Assoc. 2006; 229:531–34. 10.2460/javma.229.4.531 16910851

[39] Ruple A, Jones M, Simpson M, Page R. The Golden Retriever Lifetime Study: Assessing factors associated with owner compliance after the first year of enrollment. J Vet Intern Med. 2021; 35:142–49. 10.1111/jvim.15921 33191623 PMC7848307

[40] da Costa JP, Vitorino R, Silva GM, Vogel C, Duarte AC, Rocha-Santos T. A synopsis on aging-Theories, mechanisms and future prospects. Ageing Res Rev. 2016; 29:90–112. 10.1016/j.arr.2016.06.005 27353257 PMC5991498

[41] Piedrafita G, Keller MA, Ralser M. The Impact of Non-Enzymatic Reactions and Enzyme Promiscuity on Cellular Metabolism during (Oxidative) Stress Conditions. Biomolecules. 2015; 5:2101–22. 10.3390/biom5032101 26378592 PMC4598790

[42] Jang JY, Blum A, Liu J, Finkel T. The role of mitochondria in aging. J Clin Invest. 2018; 128:3662–70. 10.1172/JCI120842 30059016 PMC6118639

[43] Powell SR, Wang P, Divald A, Teichberg S, Haridas V, McCloskey TW, Davies KJ, Katzeff H. Aggregates of oxidized proteins (lipofuscin) induce apoptosis through proteasome inhibition and dysregulation of proapoptotic proteins. Free Radic Biol Med. 2005; 38:1093–101. 10.1016/j.freeradbiomed.2005.01.003 15780767

[44] Wong JM, Collins K. Telomere maintenance and disease. Lancet. 2003; 362:983–88. 10.1016/S0140-6736(03)14369-3 14511933

[45] De Cecco M, Ito T, Petrashen AP, Elias AE, Skvir NJ, Criscione SW, Caligiana A, Brocculi G, Adney EM, Boeke JD, Le O, Beauséjour C, Ambati J, et al. L1 drives IFN in senescent cells and promotes age-associated inflammation. Nature. 2019; 566:73–78. 10.1038/s41586-018-0784-9 30728521 PMC6519963

[46] Simon M, Van Meter M, Ablaeva J, Ke Z, Gonzalez RS, Taguchi T, De Cecco M, Leonova KI, Kogan V, Helfand SL, Neretti N, Roichman A, Cohen HY, et al. LINE1 Derepression in Aged Wild-Type and SIRT6-Deficient Mice Drives Inflammation. Cell Metab. 2019; 29:871–85.e5. 10.1016/j.cmet.2019.02.014 30853213 PMC6449196

[47] Tatar M, Bartke A, Antebi A. The endocrine regulation of aging by insulin-like signals. Science. 2003; 299:1346–51. 10.1126/science.1081447 12610294

[48] Thomas R, Wang W, Su DM. Contributions of Age-Related Thymic Involution to Immunosenescence and Inflammaging. Immun Ageing. 2020; 17:2. 10.1186/s12979-020-0173-8 31988649 PMC6971920

[49] Ray D, Yung R. Immune senescence, epigenetics and autoimmunity. Clin Immunol. 2018; 196:59–63. 10.1016/j.clim.2018.04.002 29654845 PMC6548177

[50] Connolly SL, Nelson S, Jones T, Kahn J, Constable PD. The effect of age and sex on selected hematologic and serum biochemical analytes in 4,804 elite endurance-trained sled dogs participating in the Iditarod Trail Sled Dog Race pre-race examination program. PLoS One. 2020; 15:e0237706. 10.1371/journal.pone.0237706 32817656 PMC7444536

[51] Jahr TH, Fergestad ME, Brynildsrud O, Brun-Hansen H, Skancke E. Haematological and serum biochemical values in Norwegian sled dogs before and after competing in a 600 km race. Acta Vet Scand. 2019; 61:20. 10.1186/s13028-019-0453-5 31023353 PMC6485113

[52] American Animal Hospital Association vaccination guidelines. 2018. https://www.aaha.org/aaha-guidelines/vaccination-canine-configuration/vaccination-canine/

[53] Guide for the care and use of laboratory animals. 2011. https://grants.nih.gov/grants/olaw/guide-for-the-care-and-use-of-laboratory-animals.pdf

[54] Shiovitz-Ezra S, Leitsch S, Graber J, Karraker A. Quality of life and psychological health indicators in the national social life, health, and aging project. J Gerontol B Psychol Sci Soc Sci. 2009 (Suppl 1); 64:i30–37. 10.1093/geronb/gbn020 19204071 PMC2800813

[55] Fuchs J, Scheidt-Nave C, Hinrichs T, Mergenthaler A, Stein J, Riedel-Heller SG, Grill E. Indicators for healthy ageing--a debate. Int J Environ Res Public Health. 2013; 10:6630–44. 10.3390/ijerph10126630 24317381 PMC3881131

[56] Rockwood K, Howlett SE. Fifteen years of progress in understanding frailty and health in aging. BMC Med. 2018; 16:220. 10.1186/s12916-018-1223-3 30477486 PMC6258409

[57] Rockwood K, Blodgett JM, Theou O, Sun MH, Feridooni HA, Mitnitski A, Rose RA, Godin J, Gregson E, Howlett SE. A Frailty Index Based On Deficit Accumulation Quantifies Mortality Risk in Humans and in Mice. Sci Rep. 2017; 7:43068. 10.1038/srep43068 28220898 PMC5318852

[58] Banzato T, Franzo G, Di Maggio R, Nicoletto E, Burti S, Cesari M, Canevelli M. A Frailty Index based on clinical data to quantify mortality risk in dogs. Sci Rep. 2019; 9:16749. 10.1038/s41598-019-52585-9 31727920 PMC6856105

[59] Pyrkov TV, Avchaciov K, Tarkhov AE, Menshikov LI, Gudkov AV, Fedichev PO. Longitudinal analysis of blood markers reveals progressive loss of resilience and predicts human lifespan limit. Nat Commun. 2021; 12:2765. 10.1038/s41467-021-23014-1 34035236 PMC8149842

[60] Bellows J, Colitz CM, Daristotle L, Ingram DK, Lepine A, Marks SL, Sanderson SL, Tomlinson J, Zhang J. Defining healthy aging in older dogs and differentiating healthy aging from disease. J Am Vet Med Assoc. 2015; 246:77–89. 10.2460/javma.246.1.77 25517329

[61] Garatachea N, Pareja-Galeano H, Sanchis-Gomar F, Santos-Lozano A, Fiuza-Luces C, Morán M, Emanuele E, Joyner MJ, Lucia A. Exercise attenuates the major hallmarks of aging. Rejuvenation Res. 2015; 18:57–89. 10.1089/rej.2014.1623 25431878 PMC4340807

[62] Piccione G, Casella S, Panzera M, Giannetto C, Fazio F. Effect of moderate treadmill exercise on some physiological parameters in untrained Beagle dogs. Exp Anim. 2012; 61:511–15. 10.1538/expanim.61.511 23095814

[63] Ferasin L, Marcora S. Reliability of an incremental exercise test to evaluate acute blood lactate, heart rate and body temperature responses in Labrador retrievers. J Comp Physiol B. 2009; 179:839–45. 10.1007/s00360-009-0367-z 19455341

[64] Steiss J, Ahmad HA, Cooper P, Ledford C. Physiologic responses in healthy Labrador Retrievers during field trial training and competition. J Vet Intern Med. 2004; 18:147–51. 10.1892/0891-6640(2004)18<147:prihlr>2.0.co;2 15058763

[65] Nold JL, Peterson LJ, Fedde MR. Physiological changes in the running greyhound (Canis domesticus): influence of race length. Comp Biochem Physiol A Comp Physiol. 1991; 100:623–27. 10.1016/0300-9629(91)90380-u 1685974

[66] McKenzie EC, Jose-Cunilleras E, Hinchcliff KW, Holbrook TC, Royer C, Payton ME, Williamson K, Nelson S, Willard MD, Davis MS. Serum chemistry alterations in Alaskan sled dogs during five successive days of prolonged endurance exercise. J Am Vet Med Assoc. 2007; 230:1486–92. 10.2460/javma.230.10.1486 17504039

[67] Bell MA, Levine CB, Downey RL, Griffitts C, Mann S, Frye CW, Wakshlag JJ. Influence of endurance and sprinting exercise on plasma adiponectin, leptin and irisin concentrations in racing Greyhounds and sled dogs. Aust Vet J. 2016; 94:154–59. 10.1111/avj.12436 27113986

[68] von Pfeil DJ, Cummings BP, Loftus JP, Levine CB, Mann S, Downey RL, Griffitts C, Wakshlag JJ. Evaluation of plasma inflammatory cytokine concentrations in racing sled dogs. Can Vet J. 2015; 56:1252–56. 26663920 PMC4668826

[69] Wakshlag JJ, Stokol T, Geske SM, Greger CE, Angle CT, Gillette RL. Evaluation of exercise-induced changes in concentrations of C-reactive protein and serum biochemical values in sled dogs completing a long-distance endurance race. Am J Vet Res. 2010; 71:1207–13. 10.2460/ajvr.71.10.1207 20919909

[70] Frye CW, Mann S, Joseph JL, Hansen C, Sass B, Wakshlag JJ. Serum Biochemistry and Inflammatory Cytokines in Racing Endurance Sled Dogs With and Without Rhabdomyolysis. Front Vet Sci. 2018; 5:145. 10.3389/fvets.2018.00145 30073172 PMC6060244

[71] Aengevaeren VL, Hopman MT, Thompson PD, Bakker EA, George KP, Thijssen DH, Eijsvogels TM. Exercise-Induced Cardiac Troponin I Increase and Incident Mortality and Cardiovascular Events. Circulation. 2019; 140:804–14. 10.1161/CIRCULATIONAHA.119.041627 31401842

[72] Stavroulakis GA, George KP. Exercise-induced release of troponin. Clin Cardiol. 2020; 43:872–81. 10.1002/clc.23337 31975465 PMC7403670

[73] Suzuki K, Nakaji S, Yamada M, Totsuka M, Sato K, Sugawara K. Systemic inflammatory response to exhaustive exercise. Cytokine kinetics. Exerc Immunol Rev. 2002; 8:6–48. 12690937

[74] Yazwinski M, Milizio JG, Wakshlag JJ. Assessment of serum myokines and markers of inflammation associated with exercise in endurance racing sled dogs. J Vet Intern Med. 2013; 27:371–76. 10.1111/jvim.12046 23398265

[75] Kunst CM, Pease AP, Nelson NC, Habing G, Ballegeer EA. Computed tomographic identification of dysplasia and progression of osteoarthritis in dog elbows previously assigned OFA grades 0 and 1. Vet Radiol Ultrasound. 2014; 55:511–20. 10.1111/vru.12171 24833331

[76] Knazovicky D, Helgeson ES, Case B, Gruen ME, Maixner W, Lascelles BD. Widespread somatosensory sensitivity in naturally occurring canine model of osteoarthritis. Pain. 2016; 157:1325–32. 10.1097/j.pain.0000000000000521 26901805 PMC4866583

[77] Fülöp T, Dupuis G, Witkowski JM, Larbi A. The Role of Immunosenescence in the Development of Age-Related Diseases. Rev Invest Clin. 2016; 68:84–91. 27103044

[78] Mahmoudi S, Xu L, Brunet A. Turning back time with emerging rejuvenation strategies. Nat Cell Biol. 2019; 21:32–43. 10.1038/s41556-018-0206-0 30602763 PMC7653017

[79] Campisi J. Senescent cells, tumor suppression, and organismal aging: good citizens, bad neighbors. Cell. 2005; 120:513–22. 10.1016/j.cell.2005.02.003 15734683

[80] Ovadya Y, Landsberger T, Leins H, Vadai E, Gal H, Biran A, Yosef R, Sagiv A, Agrawal A, Shapira A, Windheim J, Tsoory M, Schirmbeck R, et al. Impaired immune surveillance accelerates accumulation of senescent cells and aging. Nat Commun. 2018; 9:5435. 10.1038/s41467-018-07825-3 30575733 PMC6303397

[81] Franceschi C, Capri M, Monti D, Giunta S, Olivieri F, Sevini F, Panourgia MP, Invidia L, Celani L, Scurti M, Cevenini E, Castellani GC, Salvioli S. Inflammaging and anti-inflammaging: a systemic perspective on aging and longevity emerged from studies in humans. Mech Ageing Dev. 2007; 128:92–105. 10.1016/j.mad.2006.11.016 17116321

[82] Palmer DB. The effect of age on thymic function. Front Immunol. 2013; 4:316. 10.3389/fimmu.2013.00316 24109481 PMC3791471

[83] Pawelec G. Immunosenenescence: role of cytomegalovirus. Exp Gerontol. 2014; 54:1–5. 10.1016/j.exger.2013.11.010 24291068

[84] Fülöp T, Larbi A, Pawelec G. Human T cell aging and the impact of persistent viral infections. Front Immunol. 2013; 4:271. 10.3389/fimmu.2013.00271 24062739 PMC3772506

[85] Day MJ. Ageing, immunosenescence and inflammageing in the dog and cat. J Comp Pathol. 2010 (Suppl 1); 142:S60–69. 10.1016/j.jcpa.2009.10.011 20005526

[86] Pawelec G, Barnett Y, Forsey R, Frasca D, Globerson A, McLeod J, Caruso C, Franceschi C, Fülöp T, Gupta S, Mariani E, Mocchegiani E, Solana R. T cells and aging, January 2002 update. Front Biosci. 2002; 7:d1056–183. 10.2741/a831 11991846

[87] Muller GC, Gottlieb MG, Luz Correa B, Gomes Filho I, Moresco RN, Bauer ME. The inverted CD4:CD8 ratio is associated with gender-related changes in oxidative stress during aging. Cell Immunol. 2015; 296:149–54. 10.1016/j.cellimm.2015.05.006 26051633

[88] Wikby A, Maxson P, Olsson J, Johansson B, Ferguson FG. Changes in CD8 and CD4 lymphocyte subsets, T cell proliferation responses and non-survival in the very old: the Swedish longitudinal OCTO-immune study. Mech Ageing Dev. 1998; 102:187–98. 10.1016/s0047-6374(97)00151-6 9720651

[89] Greeley EH, Ballam JM, Harrison JM, Kealy RD, Lawler DF, Segre M. The influence of age and gender on the immune system: a longitudinal study in Labrador Retriever dogs. Vet Immunol Immunopathol. 2001; 82:57–71. 10.1016/s0165-2427(01)00336-1 11557294

[90] Holder A, Mella S, Palmer DB, Aspinall R, Catchpole B. An Age-Associated Decline in Thymic Output Differs in Dog Breeds According to Their Longevity. PLoS One. 2016; 11:e0165968. 10.1371/journal.pone.0165968 27824893 PMC5100965

[91] Goulart MR, Pluhar GE, Ohlfest JR. Identification of myeloid derived suppressor cells in dogs with naturally occurring cancer. PLoS One. 2012; 7:e33274. 10.1371/journal.pone.0033274 22428007 PMC3302813

[92] Sherger M, Kisseberth W, London C, Olivo-Marston S, Papenfuss TL. Identification of myeloid derived suppressor cells in the peripheral blood of tumor bearing dogs. BMC Vet Res. 2012; 8:209. 10.1186/1746-6148-8-209 23110794 PMC3557223

[93] Hall JA, Chinn RM, Vorachek WR, Gorman ME, Jewell DE. Aged Beagle dogs have decreased neutrophil phagocytosis and neutrophil-related gene expression compared to younger dogs. Vet Immunol Immunopathol. 2010; 137:130–35. 10.1016/j.vetimm.2010.05.002 20605222

[94] Allison LN, Jaffey JA, Bradley-Siemens N, Tao Z, Thompson M, Backus RC. Immune function and serum vitamin D in shelter dogs: A case-control study. Vet J. 2020; 261:105477. 10.1016/j.tvjl.2020.105477 32741494

[95] Prpar Mihevc S, Majdič G. Canine Cognitive Dysfunction and Alzheimer’s Disease - Two Facets of the Same Disease? Front Neurosci. 2019; 13:604. 10.3389/fnins.2019.00604 31249505 PMC6582309

[96] Salvin HE, McGreevy PD, Sachdev PS, Valenzuela MJ. Under diagnosis of canine cognitive dysfunction: a cross-sectional survey of older companion dogs. Vet J. 2010; 184:277–81. 10.1016/j.tvjl.2009.11.007 20005753

[97] Landsberg GM, Deporter T, Araujo JA. Clinical signs and management of anxiety, sleeplessness, and cognitive dysfunction in the senior pet. Vet Clin North Am Small Anim Pract. 2011; 41:565–90. 10.1016/j.cvsm.2011.03.017 21601747

[98] Takeuchi T, Harada E. Age-related changes in sleep-wake rhythm in dog. Behav Brain Res. 2002; 136:193–99. 10.1016/s0166-4328(02)00123-7 12385805

[99] Siwak CT, Tapp PD, Milgram NW. Effect of age and level of cognitive function on spontaneous and exploratory behaviors in the beagle dog. Learn Mem. 2001; 8:317–25. 10.1101/lm.41701 11773431 PMC311391

[100] Siwak CT, Tapp PD, Zicker SC, Murphey HL, Muggenburg BA, Head E, Cotman CW, Milgram NW. Locomotor activity rhythms in dogs vary with age and cognitive status. Behav Neurosci. 2003; 117:813–24. 10.1037/0735-7044.117.4.813 12931965

[101] Siwak CT, Murphey HL, Muggenburg BA, Milgram NW. Age-dependent decline in locomotor activity in dogs is environment specific. Physiol Behav. 2002; 75:65–70. 10.1016/s0031-9384(01)00632-1 11890954

[102] Turcsán B, Wallis L, Berczik J, Range F, Kubinyi E, Virányi Z. Individual and group level personality change across the lifespan in dogs. Sci Rep. 2020; 10:17276. 10.1038/s41598-020-74310-7 33057125 PMC7560605

[103] Vikartovska Z, Farbakova J, Smolek T, Hanes J, Zilka N, Hornakova L, Humenik F, Maloveska M, Hudakova N, Cizkova D. Novel Diagnostic Tools for Identifying Cognitive Impairment in Dogs: Behavior, Biomarkers, and Pathology. Front Vet Sci. 2021; 7:551895. 10.3389/fvets.2020.551895 33521072 PMC7843503

[104] Kabadayi C, Bobrowicz K, Osvath M. The detour paradigm in animal cognition. Anim Cogn. 2018; 21:21–35. 10.1007/s10071-017-1152-0 29234898 PMC5756264

[105] Cummings BJ, Head E, Ruehl W, Milgram NW, Cotman CW. The canine as an animal model of human aging and dementia. Neurobiol Aging. 1996; 17:259–68. 10.1016/0197-4580(95)02060-8 8744407

[106] Satou T, Cummings BJ, Head E, Nielson KA, Hahn FF, Milgram NW, Velazquez P, Cribbs DH, Tenner AJ, Cotman CW. The progression of beta-amyloid deposition in the frontal cortex of the aged canine. Brain Res. 1997; 774:35–43. 10.1016/s0006-8993(97)81684-8 9452189

[107] Cummings BJ, Head E, Afagh AJ, Milgram NW, Cotman CW. Beta-amyloid accumulation correlates with cognitive dysfunction in the aged canine. Neurobiol Learn Mem. 1996; 66:11–23. 10.1006/nlme.1996.0039 8661247

[108] Schmidt F, Boltze J, Jäger C, Hofmann S, Willems N, Seeger J, Härtig W, Stolzing A. Detection and Quantification of β-Amyloid, Pyroglutamyl Aβ, and Tau in Aged Canines. J Neuropathol Exp Neurol. 2015; 74:912–23. 10.1097/NEN.0000000000000230 26247394

[109] Panek WK, Gruen ME, Murdoch DM, Marek RD, Stachel AF, Mowat FM, Saker KE, Olby NJ. Plasma Neurofilament Light Chain as a Translational Biomarker of Aging and Neurodegeneration in Dogs. Mol Neurobiol. 2020; 57:3143–49. 10.1007/s12035-020-01951-0 32472519 PMC7529326

[110] Panek WK, Murdoch DM, Gruen ME, Mowat FM, Marek RD, Olby NJ. Plasma Amyloid Beta Concentrations in Aged and Cognitively Impaired Pet Dogs. Mol Neurobiol. 2021; 58:483–89. 10.1007/s12035-020-02140-9 32970242 PMC7855498

[111] Rosado B, González-Martínez A, Pesini P, García-Belenguer S, Palacio J, Villegas A, Suárez ML, Santamarina G, Sarasa M. Effect of age and severity of cognitive dysfunction on spontaneous activity in pet dogs - part 2: social responsiveness. Vet J. 2012; 194:196–201. 10.1016/j.tvjl.2012.03.023 22578689

[112] Rosado B, González-Martínez A, Pesini P, García-Belenguer S, Palacio J, Villegas A, Suárez ML, Santamarina G, Sarasa M. Effect of age and severity of cognitive dysfunction on spontaneous activity in pet dogs - part 1: locomotor and exploratory behaviour. Vet J. 2012; 194:189–95. 10.1016/j.tvjl.2012.03.025 22591786

[113] Smith BP, Litchfield CA. How well do dingoes, Canis dingo, perform on the detour task? Animal Behaviour. 2010; 80:155–62. 10.1016/j.anbehav.2010.04.017

[114] Vite CH, Head E. Aging in the canine and feline brain. Vet Clin North Am Small Anim Pract. 2014; 44:1113–29. 10.1016/j.cvsm.2014.07.008 25441628 PMC4254595

[115] Head E, Pop V, Sarsoza F, Kayed R, Beckett TL, Studzinski CM, Tomic JL, Glabe CG, Murphy MP. Amyloid-beta peptide and oligomers in the brain and cerebrospinal fluid of aged canines. J Alzheimers Dis. 2010; 20:637–46. 10.3233/JAD-2010-1397 20164551 PMC2903832

[116] González-Martínez Á, Rosado B, Pesini P, Suárez ML, Santamarina G, García-Belenguer S, Villegas A, Monleón I, Sarasa M. Plasma β-amyloid peptides in canine aging and cognitive dysfunction as a model of Alzheimer’s disease. Exp Gerontol. 2011; 46:590–96. 10.1016/j.exger.2011.02.013 21377518

[117] Youssef SA, Capucchio MT, Rofina JE, Chambers JK, Uchida K, Nakayama H, Head E. Pathology of the Aging Brain in Domestic and Laboratory Animals, and Animal Models of Human Neurodegenerative Diseases. Vet Pathol. 2016; 53:327–48. 10.1177/0300985815623997 26869150

[118] Dewey CW, Davies ES, Xie H, Wakshlag JJ. Canine Cognitive Dysfunction: Pathophysiology, Diagnosis, and Treatment. Vet Clin North Am Small Anim Pract. 2019; 49:477–99. 10.1016/j.cvsm.2019.01.013 30846383

[119] Mattsson N, Andreasson U, Zetterberg H, Blennow K, and Alzheimer’s Disease Neuroimaging Initiative. Association of Plasma Neurofilament Light With Neurodegeneration in Patients With Alzheimer Disease. JAMA Neurol. 2017; 74:557–66. 10.1001/jamaneurol.2016.6117 28346578 PMC5822204

[120] de Wolf F, Ghanbari M, Licher S, McRae-McKee K, Gras L, Weverling GJ, Wermeling P, Sedaghat S, Ikram MK, Waziry R, Koudstaal W, Klap J, Kostense S, et al. Plasma tau, neurofilament light chain and amyloid-β levels and risk of dementia; a population-based cohort study. Brain. 2020; 143:1220–32. 10.1093/brain/awaa054 32206776 PMC7174054

[121] Vijg J, Dong X. Pathogenic Mechanisms of Somatic Mutation and Genome Mosaicism in Aging. Cell. 2020; 182:12–23. 10.1016/j.cell.2020.06.024 32649873 PMC7354350

[122] Horvath S, Raj K. DNA methylation-based biomarkers and the epigenetic clock theory of ageing. Nat Rev Genet. 2018; 19:371–84. 10.1038/s41576-018-0004-3 29643443

[123] Putin E, Mamoshina P, Aliper A, Korzinkin M, Moskalev A, Kolosov A, Ostrovskiy A, Cantor C, Vijg J, Zhavoronkov A. Deep biomarkers of human aging: Application of deep neural networks to biomarker development. Aging (Albany NY). 2016; 8:1021–33. 10.18632/aging.100968 27191382 PMC4931851

[124] Petkovich DA, Podolskiy DI, Lobanov AV, Lee SG, Miller RA, Gladyshev VN. Using DNA Methylation Profiling to Evaluate Biological Age and Longevity Interventions. Cell Metab. 2017; 25:954–60.e6. 10.1016/j.cmet.2017.03.016 28380383 PMC5578459

[125] Stubbs TM, Bonder MJ, Stark AK, Krueger F, von Meyenn F, Stegle O, Reik W, and BI Ageing Clock Team. Multi-tissue DNA methylation age predictor in mouse. Genome Biol. 2017; 18:68. 10.1186/s13059-017-1203-5 28399939 PMC5389178

[126] Gorgoulis V, Adams PD, Alimonti A, Bennett DC, Bischof O, Bishop C, Campisi J, Collado M, Evangelou K, Ferbeyre G, Gil J, Hara E, Krizhanovsky V, et al. Cellular Senescence: Defining a Path Forward. Cell. 2019; 179:813–27. 10.1016/j.cell.2019.10.005 31675495

[127] Di Micco R, Krizhanovsky V, Baker D, d’Adda di Fagagna F. Cellular senescence in ageing: from mechanisms to therapeutic opportunities. Nat Rev Mol Cell Biol. 2021; 22:75–95. 10.1038/s41580-020-00314-w 33328614 PMC8344376

[128] Tchkonia T, Zhu Y, van Deursen J, Campisi J, Kirkland JL. Cellular senescence and the senescent secretory phenotype: therapeutic opportunities. J Clin Invest. 2013; 123:966–72. 10.1172/JCI64098 23454759 PMC3582125

[129] Vijg J. From DNA damage to mutations: All roads lead to aging. Ageing Res Rev. 2021; 68:101316. 10.1016/j.arr.2021.101316 33711511 PMC10018438

[130] Speakman JR. Body size, energy metabolism and lifespan. J Exp Biol. 2005; 208:1717–30. 10.1242/jeb.01556 15855403

[131] Bartke A. Somatic growth, aging, and longevity. NPJ Aging Mech Dis. 2017; 3:14. 10.1038/s41514-017-0014-y 28970944 PMC5622030

[132] Kazazian HH Jr, Moran JV. Mobile DNA in Health and Disease. N Engl J Med. 2017; 377:361–70. 10.1056/NEJMra1510092 28745987 PMC5980640

[133] Nishihara H. Transposable elements as genetic accelerators of evolution: contribution to genome size, gene regulatory network rewiring and morphological innovation. Genes Genet Syst. 2020; 94:269–81. 10.1266/ggs.19-00029 31932541

[134] Deininger PL, Batzer MA. Mammalian retroelements. Genome Res. 2002; 12:1455–65. 10.1101/gr.282402 12368238

[135] Thompson MJ, vonHoldt B, Horvath S, Pellegrini M. An epigenetic aging clock for dogs and wolves. Aging (Albany NY). 2017; 9:1055–68. 10.18632/aging.101211 28373601 PMC5391218

[136] Wang T, Ma J, Hogan AN, Fong S, Licon K, Tsui B, Kreisberg JF, Adams PD, Carvunis AR, Bannasch DL, Ostrander EA, Ideker T. Quantitative Translation of Dog-to-Human Aging by Conserved Remodeling of the DNA Methylome. Cell Syst. 2020; 11:176–85.e6. 10.1016/j.cels.2020.06.006 32619550 PMC7484147

[137] Karin O, Agrawal A, Porat Z, Krizhanovsky V, Alon U. Senescent cell turnover slows with age providing an explanation for the Gompertz law. Nat Commun. 2019; 10:5495. 10.1038/s41467-019-13192-4 31792199 PMC6889273

[138] van Deursen JM. The role of senescent cells in ageing. Nature. 2014; 509:439–46. 10.1038/nature13193 24848057 PMC4214092

[139] Kohli J, Wang B, Brandenburg SM, Basisty N, Evangelou K, Varela-Eirin M, Campisi J, Schilling B, Gorgoulis V, Demaria M. Algorithmic assessment of cellular senescence in experimental and clinical specimens. Nat Protoc. 2021; 16:2471–98. 10.1038/s41596-021-00505-5 33911261 PMC8710232

