# Supplementary material for: Development of infrastructure for a systemic multidisciplinary approach to study aging in retired sled dogs
Source: Aging (Albany NY). 2021 Sep 28;13(18):21814–37. doi: 10.18632/aging.203600 (PMC8507265; doi:10.18632/aging.203600)
Supplement: Supplementary Material [file aging-13-203600-s001.pdf]

# Development of infrastructure for a systemic multidisciplinary approach to study aging in retired sled dogs

Daria I. Fleyshman, Joseph J. Wakshlag, Heather J. Huson, John P. Loftus, Natasha J. Olby, Leonid Brodsky, Andrei V. Gudkov, and Ekaterina L. Andrianova

# Supplementary Materials

# Table of Contents

*S*upplementary Figures ........ 3

Standard Operating Procedures ........ 5

I. Afternoon (PM) Playtime Protocol ........ 5

II. Treadmill Exercise Testing........ 99

III. Cognitive Testing ........ 12

IV. Coding Cognitive Testing Videos ........ 16

V. Detour ("V") Behavior Test ........ 22

VI. Necropsy Tissue Sample Collection Protocol ........ 27

# Supplementary Figures

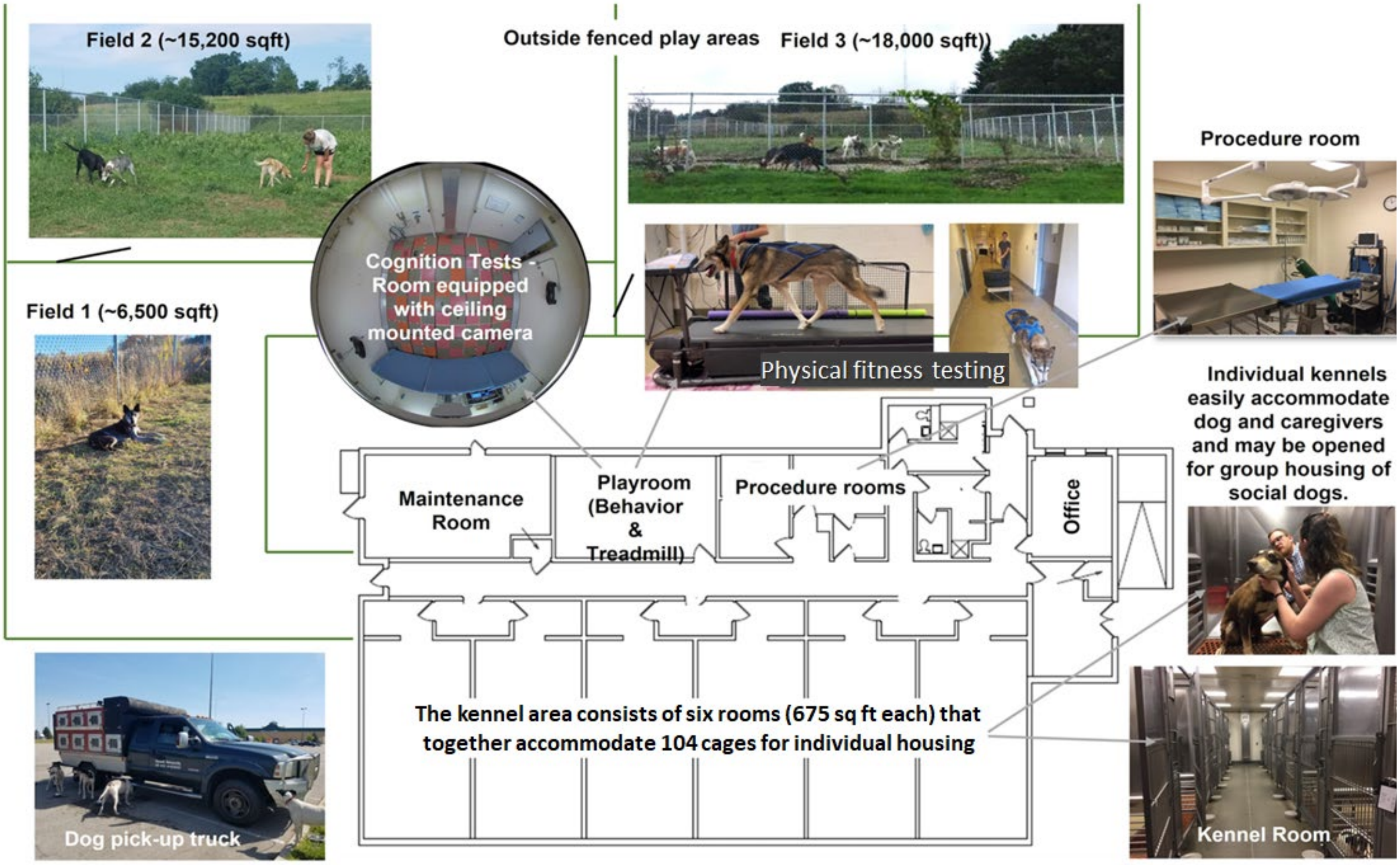


**Supplementary Figure 1.** Illustration of the Vaika, Inc. sled dog kennel and research facility at Cornell University, College of Veterinary Medicine. See main text for additional descriptions of each area and its use.

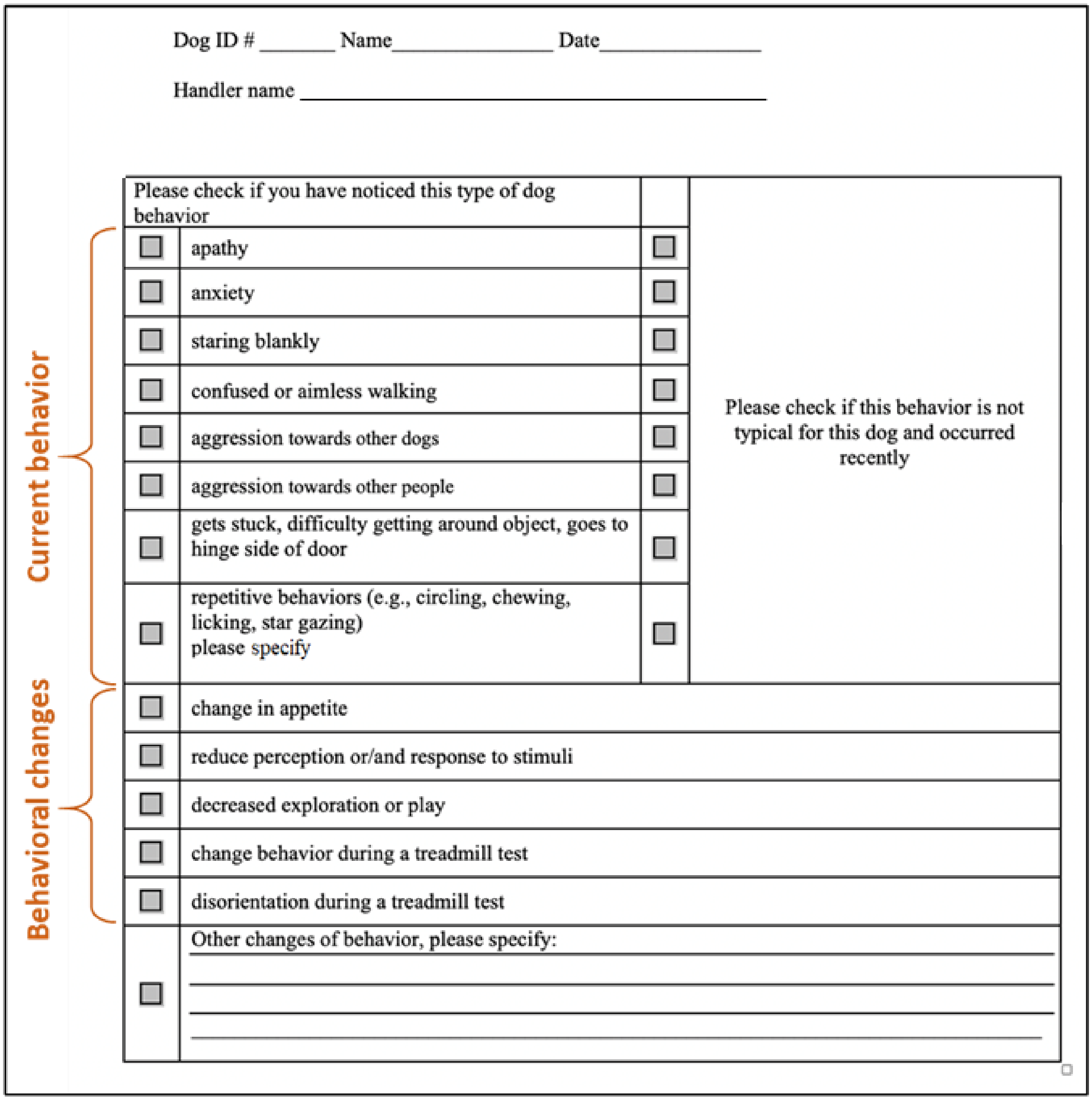

Dog ID # _______ Name_______________ Date_______________

Handler name ________________________________________

| | Please check if you have noticed this type of dog behavior | | |
|---|---|---|---|
| ☐ | apathy | ☐ | Please check if this behavior is not typical for this dog and occurred recently |
| ☐ | anxiety | ☐ | |
| ☐ | staring blankly | ☐ | |
| ☐ | confused or aimless walking | ☐ | |
| ☐ | aggression towards other dogs | ☐ | |
| ☐ | aggression towards other people | ☐ | |
| ☐ | gets stuck, difficulty getting around object, goes to hinge side of door | ☐ | |
| ☐ | repetitive behaviors (e.g., circling, chewing, licking, star gazing) please specify | ☐ | |
| ☐ | change in appetite | | |
| ☐ | reduce perception or/and response to stimuli | | |
| ☐ | decreased exploration or play | | |
| ☐ | change behavior during a treadmill test | | |
| ☐ | disorientation during a treadmill test | | |
| ☐ | Other changes of behavior, please specify: | | |



**Supplementary Figure 2.** Questionnaire for assessment of dog behaviors related to cognitive function. Questionnaires are completed every nine months. The questionnaire is completed for each dog by 5-11 handlers that regularly interact with the dog (e.g., feeding, participating in outdoor and indoor enrichment, working with the dog during treadmill testing, and handling the dogs during veterinary visits). A dog is scored as displaying a behavioral sign if it is reported by ≥50% of the responding handlers.

# Standard Operating Procedures

## I. Afternoon (PM) Playtime Protocol

**1. Before Playtime:**

- Check all pens before beginning playtime. Make sure the back door is unlocked (if the door is locked, use the key from the main entrance. See AARP Module II Training.)
- Make sure the door jamb is in the proper position and the door leading to the playtime pens ***is propped open***
- Make sure yard tools are upright and out of the way
- Check for and remove any loose garbage and/or potential ingestible hazards
- Take lids off all three trash cans and place upright behind the trash cans out of reach of the dogs
- Check for any holes dug by the dogs during prior playtime and fill in as needed
- Check for any holes in the fence and/or loose/hanging metal pieces that could be potentially dangerous for the dogs
- Extend all black collapsible gates halfway out (this makes it easier and more efficient to close quickly once a group has entered their assigned pen)
- Make sure the black collapsible gate behind the Vaika shed is closed around the equipment entrance gate in the closed pen (any yard tools that are potentially dangerous and/or ingestible hazards are kept behind this gate)
- Be sure that the middle gate is closed, and the black collapsible gate is opened halfway (not in the way of the dogs' trafficking area)
- ***Check over the enrichment sheets from the previous playtime shift for any written comments and/or concerns noted by staff or students***
- ***Check the AARP Message Board for any written notes left by staff and husbandry (see AARP Module II Training)***
- Make sure all water buckets *are full and not placed directly in a corner or alongside a fence*

**2. Letting groups out:**

- Check the white boards in the anterooms to confirm assigned pens (see AARP Module II Training). The three open play areas are separated by fences and are referred to as the far, middle and close pens. Playtime pen assignments are rotated monthly by staff. Each anteroom cabinet has two door jambs to be used during playtime.
- Close and completely lock the gate in the hallway behind room 3. These playtime gates are to remain closed for the duration of a room's playtime. Both the room and anteroom doors are to be propped open with door jambs found in the anteroom cabinet.
- The outdoor pens are filled in a set order: first, the far pen, second, the middle pen, and third the close pen.
- Before letting a group out, double check the white boards in the anterooms for color coded assigned play pens. Each dog's kennel has a colored ribbon corresponding to their assigned pen and group written on the anteroom whiteboards. ***NEVER mix play groups; if you're not sure whether or not to let a dog out, take the time to check the whiteboard and ribbon before opening a kennel door.***
- After letting dogs out, follow them outside and encourage them to go into the correct pen*. No dog should be pushed beyond their limit* (e.g., Pilot, Sway and Buddy will always move slower). Make sure the entire group has entered the pen before closing the pen gate. If you're not sure, radio to husbandry/staff/indoor teammate to double check before letting the next group out.
- In the event a dog is forgotten to be let out and is left inside after other groups have been let out, he or she ***MUST remain inside until all other dogs are back in after their allotted time outside. Once all groups are in, the forgotten dog can be let out along with its group and/or a "buddy." Dogs should never be carried or walked on leash through other groups during playtime.***

  <u>*Student, staff and dog safety are always the top priority*</u>*.*
- Once you have confirmed with staff/husbandry/teammate that all dogs are out, completely close their pen gate and then completely close their black collapsible gate. When these two gates are closed, you can radio to husbandry/staff/teammate to let out the next group out. Make sure the middle pen gate is open **BEFORE** you call for the next group.
- Encourage the middle group into their pen once you have confirmed with husbandry/staff/teammate that all dogs are out, completely close the middle pen gate, and completely close the black collapsible gate. Once these two gates have

been closed, radio to husbandry/staff/teammate to let the close pen group out. Once all dogs are confirmed to be in the close pen, remove the door jamb and close the door to the building. ***The door jamb is magnetic, it should be placed at least above head height on the building's door.***

- ***Once all dogs are out and the door to the building is closed, set your timer to 20-30 minutes.***

**3. Once all dogs are out:**

- Use the poop scoopers to begin picking up any feces overlooked from the prior playtime or already left by the group. ***Begin with the far pen and make your way to the middle and lastly the close pen. This keeps the pens as clean as possible*** and is the most efficient method. In doing so, this allows you more time to play and socialize with the dogs.
- When entering any pen, first open the black collapsible gate and then immediately close it behind you. Once the black gate is closed, you can enter the playtime pen. ***Whenever entering a pen, be sure that the black collapsible gates are closed (these act as a safety net for any dog that may sneak by you as you enter a pen. The use of these gates is essential in preventing fights, catching loose dogs and/or isolating dogs if necessary).***
- When entering the middle pen, the black collapsible gate should be looped around the middle pen gate to create a half circle or "crescent."
- Once you're done cleaning the middle pen, exit the same way you entered, latching the playtime pen gate and black collapsible gate completely behind you.
- After all pens are cleaned (should not take longer than 10 min with two students and/or one student plus one staff member), **socialize with the dogs**. This can't be emphasized enough! ***These dogs love human interaction (some prefer play, while others prefer to be groomed and "loved on") and they thrive with the more enrichment activities we can provide them.***
- Please note, after all pens are cleaned, this is not a time to sit down, text or socialize with another student(s). We encourage everyone to have their phone on them; however, close attention to the dogs during playtime is vital for observing dog behavior, finding injuries or masses, providing socialization/enrichment, and stopping potential dog fights before they begin (see AARP Module II Training before reporting any injuries or masses). Be observant: staff and husbandry rely on playtime monitors to identify wounds, abrasions or lameness that may have been missed or occurred during your playtime.
- In the event you need to come inside for a drink, restroom etc., radio to the husbandry/staff/teammate that you need to come in. ***<u>Dogs must be monitored at all times.</u>***

**4. Putting dogs back inside:**

- **Step 1.** When your timer goes off (after 20-30 minutes), round up the dogs in the close pen and radio to husbandry/staff/teammate that you're ready to let dogs in. *Wait until you get verbal confirmation from husbandry/staff/teammate before letting dogs in!* Once you're given confirmation that it's ok to let dogs inside, grab the doorstop and open the door as wide as possible. The doorstop can be placed on the hinges and left to prop the door open.

- **Step 2.** Once the entire group has entered their room and/or anteroom, close the room door (e.g., close the door to room 3). This prevents dogs from running back outside (this also helps the dogs to stay in their "daily routine" and not develop bad behavior).

- **Step 3**. Husbandry/staff and veteran students will begin putting dogs away in their assigned kennels. Appropriate dividers should be opened. ***Note* before opening any dividers, check the front of the kennel for any cards (Food in kennel, don't group house, etc.) and double check anteroom white boards as well. See AARP Module II Training for further elaboration***. All kennels have the dog's name, picture and playtime pen assignment indicated by colored tape. Dividers that can be opened are marked with black and yellow tape. ***Check the front of the kennel for instructions*** and double check inside the kennel to ensure there is no food/bowl before opening a divider.

*Steps 1-3 should be completed as quickly as possible so that dogs outside are not left unsupervised for too long. However, do not go so fast that you overlook kennel cards, dividers, completely closing kennel doors, etc.*
***Dog safety is one of our top priorities.***

- **Step 4**. Return outside to monitor the playtime groups remaining outside. Husbandry/staff/teammate will radio when the entire close pen group has been returned to their assigned kennels. Upon receiving this confirmation, open the

black collapsible gate to the middle pen. Once the black gate is opened and completely moved out of the way, you can let the middle group inside.

- **Step 5.** Repeat steps 1-4 for the far playtime pen.
- **Step 6**. Once the dogs in the far pen group are in their assigned kennels:
  - Check kennel doors before moving on to the next room. ***Some of the kennel doors can be difficult to close; pull hard to ensure it is securely latched shut.***
  - Fill out the enrichment sheet for each room before moving on to the next; these forms are inspected by IACUC and the USDA. You need to be as specific as possible. For example, include:
    - Weather conditions
    - Exactly what enrichment activity was done (grooming, playing, etc.)
    - Any unusual and or aggressive behavior (If there was a dog fight, include what dog(s) were involved, injuries that occurred etc. Please see dog fight protocol for further elaboration.
  - Repeat Steps ***1-6 for all rooms,*** making sure kennel doors are closed and secure, appropriate dividers are opened, and enrichment sheets are filled out in detail for each AM and PM playtime.

**Step 7**. **Walking of dogs on leash:**
After Steps 1-6 are completed for all rooms, return the metal door jamb to the shelf at the end of the hallway and take the dogs that are noted to be leashed walked outside for a walk. Dogs should be walked individually. Initial treatment sheet once this has been completed.

**Step 8**. **Cleaning after playtime:**
Once all specified dogs have been leash-walked and returned to their assigned kennels, empty the outdoor garbage bins. Replacement trash bags are located on the shelf at the end of the hallway. Reilley will make sure that trash bags are kept stocked and readily available. Garbage cans are to be emptied after every shift. New trash bags need to be placed in and tied around the trash cans. In addition, the following needs to be completed after every shift:

- Make sure all yard tools used are cleaned with the appropriate scrub brush marked *Poop only* after every shift. See "winter protocol" for appropriate cleaning methods in the winter.
- Garbage bin tops need to be securely put back on.
- The middle pen gate should be closed and the black collapsible gate folded neatly and secured to the fence.
- ***The far pen should be left open (gate left facing toward the entrance of the building, not the exit. This eliminates the potential for a dog to escape).***
- Empty all water buckets after every shift. In addition, after PM playtime on Saturdays, water buckets are to be cleaned with soap and water. Please use the appropriate scrub brush marked *H20 only*.
- Place full garbage bags removed from garbage bins in the dumpster
- Fill any holes dug during your playtime with rocks or wood bark chips
- For PM playtime, be sure to read and check off the sign off sheet at the end of the hallway. Please see PM Playtime protocol (Step 11 below) and AARP Module II Training for how to leave AARP appropriately.

**Step 9. Administering medications:**

- Some dogs are prescribed long term and/or acute treatments by CARE veterinary and technician staff. CARE veterinarians, technicians and husbandry are responsible for creating, monitoring and logging completed treatment sheets (see AARP Module II Training for further elaboration). ***Students and staff working during PM playtime are responsible for administering PM medications only as indicated on each dog's treatment sheet.***
- Treatment sheets can be found on clip boards below the whiteboards in the anterooms. When medicating dogs, do ***ONE*** dog at a time and make sure you're administering the correct medication as instructed.
- If the treatment sheet is unclear or you have any questions, please post them to the vetcare channel on slack. ***AFTER*** you have administered the medication to one dog, you must initial for the correct day and date on that dog's treatment sheet. ***Now*** you can move on to the next dog!

**Step 10. Spot clean up:**

- Once all dogs have received their medicine, all rooms and the hallway need to be checked for urine spots. In the janitor's closest you'll find the necessary cleaning supplies (mop bucket, mop, canine cleaning solution).

- Starting in from the end rooms (room 3 and room 1) and working towards the center room (room 2), check each anteroom and both kennel rooms (A and B) within each room for urine. ***Note, the entire aisle does not need to be mopped, just where dogs have urinated during playtime.*** While spot cleaning, this is an ideal time to double check treatment sheets, kennel doors, dividers, food bowls, etc. One student and/or staff member(s) should start in room 3B while the other student and/or staff member(s) starts in room 1A.
    - ***Each student and/or staff member will have their own mop bucket and mop; when you both get to room 2, cross check each other and keep spot cleaning/checking kennel doors etc.***
    - ***This "cross checking" method has proven to be extremely effective in catching another individual's mistake!***
- After spot cleaning and checking of treatment sheets, kennel doors, dividers, etc. is complete, the mop buckets need to be dumped and mops rinsed out in the sink located in the janitor's closet.
    - ***As an effort towards teamwork and a healthy, friendly work atmosphere, students and/or staff members are expected to help husbandry keep our facility and its cleaning supplies neat and orderly.***

**Step 11. Leaving AARP:**

- Read and check off all applicable actions on the PM playtime checklist located at the end of the hallway. Both students and/or staff member(s) must initial this paper.
- All rooms must be checked to ensure all lights are turned off (surgery room, pharmacy, outdoor lights, etc.) and all doors are locked. Please see AARP Training Module II for further instruction on how to leave the building appropriately.

# II. Treadmill Exercise Testing

**<u>1. Before Testing Begins</u>**

**1.1. PetPace Collar Setup and Placement**

1.1.1. For collar setup, refer to “SOP PetPace”.

1.1.2. After setup, place collars on the dogs scheduled for treadmill testing at least 1 hr prior to start of the test.

**1.2. Blood Draw Setup**

1.2.1. Cytokine assays

- Prepare 5 red top serum tubes for each dog (one for each time point). Label with dog name, date, time point, and specific time of collection.
- Collect 4-5ml whole blood into the appropriate red top serum tube at each of the following time points:
    - At “time 0” (immediately before the start of exercise)
    - 5 min post exercise
    - 2 hrs post exercise
    - 4 hrs post exercise
    - 24 hrs post exercise
- Allow collected blood to sit at room temperature for at least 30 minutes, but no more than 2 hours.
- Centrifuge at 1,000 x G for 10 minutes to separate serum.
- Draw off the serum sing a sterile pipette and aliquot into cryovials (up to 1ml per tube) for long-term storage at -80°C.

1.2.2. Lactate measurement – using a Novis lactate meter

- Insert test strip with the end having a metallic appearance inserted into the meter.
- Collect a single drop of whole blood, preferably from the jugular vein or cephalic vein (ear pinna as back-up option) and apply to the exposed end of the test strip. Record blood draw site on testing records.
- Record meter reading after the 13 second countdown.
- Time schedule for lactate measurement:
    - “Time 0” (immediately before the start of exercise)
    - 1 min post exercise

1.2.3. Cardiac troponin – using iSTAT cartridge

- Prepare iSTAT, insert cartridge according to the manufacturer’s instructions.
- Dispense 3 drops of uncoagulated whole blood onto iSTAT cartridge.
- Time schedule for troponin measurement:
    - “Time 0” (immediately before the start of exercise)
    - 5 min post exercise

**1.3. Treadmill Setup**

1.3.1. Open the excel spreadsheet “Vaika - Sled Dog Training Rating Scores>Winter Progress” containing data for dogs’ walk and trot speeds; move the spreadsheet to left hand screen so that it can be viewed at the same time as Gait4Dog.

1.3.2. Open Gait4Dog.exe on the desktop computer. Click on the login box 3 times and it will default and open the program.

- In Gait4Dog, click on the “Dog” icon in the upper left corner and search for the dog to be tested by name. Select the dog by name and this will open the desired dog’s profile.
- For data collection during treadmill testing:
    - Select the “Test” button.
    - Set parameters: (i) within the middle top box, select “Trot”; (ii) within the left box, select large dog, medium duration; (iii) for repetitions, select 3.
    - Select the “Continue to Trot“ button.
    - The 3 repetitions will be automatically collected one after the other assuming there are no footfall issues such as the dog stepping off of the side, front or back of the sensor area of the treadmill. If an error occurs, this will be seen on the computer screen and the data collection should be started again.

1.3.3. Gather supplies for treadmill testing: (i) Harnesses for dogs, and (ii) Stopwatch/timer.

**2. Performance of Treadmill Test**

Sequential steps for performing the treadmill test are listed below:

1. Record the dog's name and the date and time of treadmill testing in "Vaika - Sled Dog Training Rating Scores>Treadmill Testing" tab of the excel file.
2. Place a properly fitting harness on the dog.
3. Confirm that the Gait4Dog computer application is open to the correct dog's profile.
4. Confirm that the PetPace collar is positioned correctly on the dog and is set up to collect data.
5. Time 0 blood draw: Immediately prior to getting the dog on the treadmill, draw 4-5ml of whole blood into a red top tube for cytokine assays and apply a single drop to the test strip in the lactate meter for reading. Record lactate level.
6. Treadmill start phase: This phase should be ~2 minutes on the treadmill.
   a. Have the dog get onto the treadmill and hook the back of his harness to the lines behind the treadmill. Use the line directly behind the dog unless you notice the dog tends to trot on a particular side of the treadmill; in that case, the opposing line can be used instead to help correct this and keep the dog towards the center of the treadmill.
   b. Start the treadmill using the "Start" button on the treadmill control panel.
   c. Increase the speed to the dog's individually identified "Walk" speed.
   d. Increase the incline to 5%.
   e. Increase the speed to the dog's individually identified "Trot" speed. Adjust the tugline at the back of the harness if necessary to keep the dog in the center of the treadmill. Steps a-e should take less than 2 minutes.
7. Treadmill test phase:
   a. Continuing from step 6 above without stopping the treadmill, start the stopwatch as soon as the dog is comfortably trotting at his identified speed.
   b. Upon reaching 1 min at trot, start to collect Gait4Dog treadmill data using the prescribed settings.
   c. Upon reaching 15 min at trot, start a 2$^{nd}$ collection of Gait4Dog treadmill data using the prescribed settings.
   d. Upon reaching 20 min at trot, select "Stop" to gradually slow the treadmill to a stop.
   e. Reward the dog, remove the dog from the treadmill, and offer water.
8. Immediately after the treadmill stops, manually determine dog's heartrate.
9. At 1 min post-exercise (after treadmill stops): 2$^{nd}$ lactate blood draw using single drop of whole blood for the lactate meter.
10. At 2 min post-exercise: collect a 2$^{nd}$ manual heartrate.
11. At 5 min post-exercise: 2$^{nd}$ blood draw of 4-5ml whole blood into red top tube for cytokine and troponin analyses.
12. At 35-60 min post-exercise: Spin down cytokine tubes Pre-0 and Post-5 min. Transfer serum to cyrovials and freeze.
13. At 2 hr post-exercise: 3$^{rd}$ blood draw of 4-5ml whole blood into red top tube for cytokine analysis.
14. At– 2.5-3 hr post-exercise: Spin down cytokine tube Post 2hr. Transfer serum to cryovials and freeze.
15. At 4 hr post-exercise: 4$^{th}$ blood draw of 4-5 ml whole blood into red top tube for cytokine analysis.
16. Remove the Petpace collar at the time of the 4 hr cytokine blood draw.
17. At 4.5-5 hr post-exercise: Spin down cytokine tube Post 4 hr. Transfer serum to cryovials and freeze.
18. At 24 hr post-exercise: 5$^{th}$ blood draw of 4-5 ml whole blood into red top tube for cytokine analysis.
19. At 24.5-25 hr post-exercise: Spin down cytokine tube Post 24 hr. Transfer serum to cryovials and freeze.

**3. Troubleshooting**

Any deviation from the prescribed routine should be indicated in the data recording in red font and described in writing in the note section.

If a dog stops on the treadmill during the 20 min trot test, proceed as follows:

- Encourage dog to continue before stopping the treadmill itself if the dog appears mostly hesitant and tired or is placing feet on the side panels.

- Pull the emergency stop cord on the treadmill control panel to stop the treadmill quickly if the dog falls, has any health concerns with proceeding test, stops completely without any interest in restarting, or will not keep its feet off of the side panels.
    - Stop the 20 min timer and take note of total time completed by the dog on the treadmill.
- If there are no injuries or health concerns for the dog, allow the dog to stand normally and confidently again on the stopped treadmill and restart at a walk immediately. Continue as with the initial start, increasing the incline to 5%. Depending upon the dog and situation, increase the speed to the dog's trot speed immediately after increasing the incline.
    - Restart the 20 min timer for the remainder of the time left once the dog is trotting again.
- If the dog cannot be restarted, continues to stop after the new start, or there are any health concerns, do not proceed further - STOP the test. Address any health concerns as necessary. Otherwise, record the total time completed at trot and proceed with blood draws and heartrate collections following the same schedule of post-exercise time points. Note in the testing records the dog's behavior and what happened.

## III. Cognitive Testing

**1. Before Testing Begins**

**1.1. Novel Person Acquisition and Handling**

- Potential "Novel People" for use in open field cognitive testing can be contacted using information in the "Potential Novel Persons for the Future" file in Vaika Project Cornell->Behavior Testing->Misc. Behavior Items->novel people, reordering & such-> Potential Novel Persons for the Future file. This file contains information of previous Novel People, including the dogs they saw and their contact email addresses. It is also possible to find new volunteers by sending listserv emails to the vet school (John + Heather can help with this). **Begin this process well in advance of testing day; about 3-4 weeks ahead** should be enough to begin a schedule. Get creative! If things get dire, rope someone's kids into it. In the folder "novel people, reordering & such" there is an excel sheet "9 mo. Behavior scheduling (dogs + ppl)" that provides a framework to start scheduling both dogs and people.
- Keep Novel People novel for the dogs by never allowing a dog to see a person they have already seen (i.e., if Swag the dog had Melissa as a novel person for her NPT at the 9-month timepoint, Swag may not have Melissa for her 18 month NPT. Similarly, if Mark plays with Stinka the dog after one of his sessions, he may not be Stinka's NPT person).
- Send reminder emails to participating Novel People the day before they are scheduled to come in to reduce the risk of no-shows. Ask student helpers to do this or prepare in advance.
- When you meet a Novel Person for the first time, be sure to give them a complete run down of what is expected, where they will go, and make sure they know how to dress for the test. Reiterate how important it is to have a new gown and new shoe covers EVERY time they step into the testing room and make sure they are familiar with your handler and come up with a system with your handler so that they can call the Novel Person without letting the dog see them before the test (walkie talkies work well).

**1.2. Preparation for Running Open Field Tests**

- Become familiar with the protocol and ask to do a dry run with pet dogs (see the dry run folder in Box, used John's dog Lily, etc.). It is possible to get each dog's 4 tests (OFT, NOT, NPT & MT) finished in 30 minutes, but you must mop in between each test (all of the floor and pee/poop spots), set up apparatus, and coordinate with the handler and Novel People.
- Carefully maintain the Google Sheet: 'Vaika Behavior Coding Log Checklist' (https://docs.google.com/spreadsheets/d/1s3JLCT1EWr3g5h9szsQmIjHfm_rvb6tAD-wJtpgHgvc/edit#gid=0). All behavior coders are to use this checklist to keep track of completed coded videos, sex + age, upcoming testing timepoints, miscellaneous info and strikethrough deceased dogs and black bar out their next timepoints so there is continuity (and you won't be looking for a deceased dog's video). **Please** monitor the VetCare channel for males that get neutered and note the date of neuter in the "sex" column.

**1.3. Computer Setup - Observer**

1. Setup Zoom meeting on office computers. Retrieve the Behavior External Hard Drive from the bottom drawer of the Vaika file cabinet in the office.
2. Enter the "Testing Room" and ensure that lights, computer, and camera system are turned on. Ensure the camera system microphone cable is inserted in the microphone jack on the computer. Log onto the computer using the HusonLab id & password. If the microphone cable was unplugged, follow any computer prompt windows that appear after plugging in the cable.
3. On the desktop, double-click the Media Recorder icon (red with white camera icon) to turn on the camera. Once the program opens, select "File", then "Preferences", then "N-link settings", then "Test Connection". Make sure the window reads "Connected". Click "Ok".
4. In the Media Recorder program, select "File", then "Video Settings…". In the pop-up window, ensure the check box is filled next to "Use maximum recording time". Change the time to "00:03:00" to record for 3 minutes. Click "Apply". Drag the Media Recorder window to the second screen.
5. Accept Zoom meeting request and enable screen share. Share "Screen 2".
6. Mute the computer's sound.
7. Open two separate Notepad windows. Keep both windows in Screen 1 (with PetPace website window).

a. In one window, type “Camera error - retrieve dog”. If during testing, the camera freezes, slide the “Camera error” window to screen 2 so the dog can be retrieved to stop testing. **The dog must be removed from the room before attempting ANY troubleshooting**.
b. In the other Notepad window, type “Lunch break”. When stopping for lunch, slide the “Lunch break” window to screen 2 and close the Media Recorder window. When finished with lunch, slide back to screen 1 (or delete) and repeat steps 5-6.

8. Ensure all three walkie-talkies are turned on and set to the same channel. Leave one walkie-talkie in the office, one in the Testing Room, and the third goes with the Handler.

**1.4. Room Cleaning - Handler**

1. If any clumps of fur or large debris is present on testing area floor, vacuum it using the black and red vacuum found on the wall near the treadmill in the Testing Room.
2. Place the floor mat outside the door, propped against the wall.
3. Grab the trash can from anteroom 2, two red chairs, the spiky + clear floor mat and the white shoe cover holder. Place the white spiky mat just outside the door to the playroom as a “semi-clean” area. Grab both red chairs and place the first two legs of the chairs on the mat facing toward each other with one chair open and use the other to hold the isolation gowns and shoe covers (the Novel Person will sit in the free chair as they put their isolation gown and shoe covers on). Use the trash can to throw away the shoe covers and gown after each test. Novel Person is to wear a new gown and new shoe covers every time they enter the playroom.
4. Place the bins of blue shoe covers and yellow isolation gowns outside the Testing Room door. Blue shoe covers can be found in the clear plastic tub on top of the playroom computer cabinet by the treadmill. Shoe covers should be worn in the testing room once the floor has been mopped. If there is a need to exit the Testing Room after mopping, new shoe covers should be put on before re-entering the room.

**2. Performance of Tests**

Four open field tests will be performed consecutively on a single day in the following sequence for each dog: a true open field test with an empty room (“OFT”), followed by open field tests with a novel object (“NOT”), a novel person (“NPT”), or a mirror (“MT”) introduced.

2.1. Test 1: Open Field Test “OFT”

1. The hallway/ door signs should be placed to indicate that testing is in progress and persons in the AARP building need to remain quiet.
2. Phones should be placed on silent.
3. In the Media Recorder program, on the bottom right side are two boxes labeled “File Location” and “File Base Name”. Next to the “File Location” box, ensure it says “C:\Users\husonlab\Desktop\Recordings”. In the “File Base Name” box, enter the dog’s name, date, and “OFT” (ex: Trapper_01.25.19_OFT). Ensure Video Settings are set to record for 3 minutes (Step 6 of Computer Setup).
4. Two people are required to perform the testing, herein referred to as Handler and Observer.
5. The Handler will take the walkie-talkie from the office and one leash from the wall hooks near the desk in the Testing Room and go to collect the dog for testing.
6. The Observer will update the whiteboard with the dog’s name, and “1”. Write very BIG and ensure it can be read in the video feed on the computer.
7. The Observer will roll the mobile divider into place in the Testing Room, and then place the red plastic chair against the wall behind the mobile divider to prevent dogs from pushing in the mobile divider. When ready, the Observer will notify the Handler with the walkie talkie OR via text/Slack.
8. The Handler will take the dog to the “Close Pen” to relieve itself. If the dog has not done anything in 3 minutes the Handler may proceed to the Testing Room to begin testing.
9. The Observer will begin the recording through the MediaRecorder program (click the red record button “Start Recording” at the bottom of the screen) as soon as the dog and Handler enter the room.
10. The Handler will release the dog into the room without the Handler entering the room and quickly close the door. The Handler can then proceed to the office to wait for the duration of the test and to watch the testing via Zoom.
11. The Observer will remain in the Testing Room behind the dividers while the test is underway. It is essential that the Observer remain perfectly quiet and still during this time.

12. After 3 minutes, the testing will end and the recording will automatically stop. The Handler will return to the Testing Room to collect the dog after the video flashes to indicate recording has stopped (or are texted to retrieve). Please open the door slowly as some dogs will crowd the door and get hit if you open it too quickly.
13. The Handler will close the gates on either side of the Testing Room in the hallway. They will then open to the door to the Testing Room. If the dog willingly exits, the Handler will leash the dog in the hallway. If the dog does not exit the room on their own, the Handler will put on shoe covers, enter the room quickly to retrieve the dog by the collar. The leash may be clipped on the collar while in the hall. The Handler should refrain from talking/interacting with the dog aside from ushering them out of the Testing Room.
14. The Handler will then take the dog out to the "Close Pen" while the observer resets the room.

2.2. Test 2: Novel Object Test "NOT"

1. The Observer will obtain a stuffed toy turtle, a yellow cone, and a red Kong from the bottom drawer of the Testing room desk.
2. The Observer will enter the testing area wearing shoe covers by moving the mobile divider. They will place the turtle on the spot labeled "T" on the floor, the cone on "C", and the Kong on "K". They will update the whiteboard with the dog's name and number "2". The Observer will exit the testing area and place the mobile divider and red chair back in place.
3. The Observer will set up a new recording. Label the "File Base Name" box in the Recorder program with the dog's name, date, and "NOT".
4. The Observer will notify the Handler that the room is ready to begin testing by walkie talkie or text/Slack.
5. Steps 9-14 of the OFT protocol will be repeated.

2.3. Test 3: Novel Person Test "NPT"

1. The Handler will notify the Novel Person via walkie-talkie that they are outside so the Novel Person can leave the office and enter the testing area wearing the isolation gown and plastic shoe covers.
2. The Observer will move the mobile divider to the testing area and will place the black chair from behind the dividers in the same spot as the Kong toy (square #2) in the testing area. The chair will be placed to be facing the wall with the door.
3. The Observer will update the white board with the number "3".
4. The Observer will remove the novel objects and place them in the travel box next to the desk.
5. The Novel Person will sit in the chair with both feet touching the floor, hands in their lap and a neutral gaze.
6. The Observer will inform the Novel Person of the following instructions: He/she must look directly at the wall in front of them without moving or looking at the dog at all. They should not react to anything the dog is doing or pay it any attention. They are not to get up until the dog exits the room.
7. The Observer will return behind the divider, placing the mobile divider back in place. The Observer will set up a new recording, labeling it with the dog's name, date, and "NPT". They will notify the Handler by walkie talkie that the testing room is ready.
8. Steps 9-14 of the OFT protocol will be repeated.

2.4. Test 4: Mirror Test "MT"

1. The Observer will set up a new recording on the computer, labeling it with the dog's name, date, and "MT". They will also update the whiteboard with the number "4".
2. There will be a mirror leaning against the wall near the desk. Bring it into the testing room and attach it to the wall that is opposite the door in the designated spot. The mirror is secured in place by placing the top edge into the upper clips, pushing up against the springs, and allowing the base of the mirror to settle into the lower clips.
3. The Observer will return behind the divider and replace the mobile divider.
4. The Observer will notify the Handler that the room is ready to begin testing by walkie talkie.
5. Repeat steps 9-14 of the OFT protocol.

2.5. After Completion of OFT, NOT, NPT and MT for a Given Dog

1. Once the dog exits the room after completion of the MT, the PetPace collar will remain on for 24 hours and the dog can be returned to its kennel by the Handler while the Observer completes the recording details.

2. The Observer will turn on the fans and update the white board for the next dog.
3. The Observer will wipe down the mirror with paper towels and Rescue cleaner from the desk area, remove the mirror from the wall, and return it to the area near the desk. Always make sure the reflective side is facing toward the wall, so dogs cannot see it when they do treadmill training.
4. The Handler may begin mopping the room as described in “Day After Behavior Testing” for the next dog.
5. The Observer will use Rescue Spray (at the Testing room desk) solution and paper towels to wipe down the chair used in the NPT.
6. When the floor is dry, the Observer will turn off the fans and proceed with testing of the next dog, beginning with Step 3 of the OFT (see Section 2.1 above).

**3. End of Testing Day**

1. The Observer will copy all recordings to the “Behavior” external hard drive and to the Box “Behavior Videos” share folder.
2. The Handler will use the blue hose and spray down the mat, the floor, walls, and divider. Mop the mat and mobile divider with the Rescue cleaner, then drape the mat over the mobile divider. Proceed with mopping the floor (see “Day After Behavior Testing” below).
3. The Handler will take the box of items from the NOT to the sink near the front door to clean them with Rescue cleaner. The objects will be placed in the rack to dry. The ‘fire hose’ turtle toy will need to be washed in a laundry cycle; all turtle toys from the day will be washed together with the white mop head after the last behavior test for the day.
4. The Observer will update the PetPace Notebook with PetPace collar id tracking/charging information, and the time of any noises heard in the Testing room during testing periods.

**4. Day After Behavior Testing Entirely Finished for All Dogs**

1. Mop floor using designated testing mops labeled with tape on the handles which can be found in the custodial closet and the white mop head. Make a 1:16 dilution of the Rescue Concentrate (8 pumps per gallon; 2 pumps per liter). Mop entire area of the floor designated for testing by the black dividers in the Testing Room, as well as the metal borders of the room and bottom sections of the dividers. Start mopping in corner “A”, because the water pools, then squeegee the water into the floor drain right away. Continue mopping towards corner “B”. Water also pools in this corner so water should be squeegeed right away. Continue mopping towards corner “C”, turn on fan (on back of fan), continue mopping towards corner “D” and back to corner “A”. Turn on fan near corner “A”. Squeegee any remaining water towards corner “A” and exit through the door with the final squeegee.
2. Once you’ve exited the Testing Room after cleaning, take a standard mop from the custodial closet to mop up any water that flowed under the door. (This does not need to be a clean mop). You will then lay down the mat you placed outside the door in front of the door.
3. To return into the room, put on blue shoe covers every time before entering the room.
4. Once the floor has no visible wet spots, turn off the fans.

# IV. Coding Cognitive Testing Videos

**1. Computer Setup – 208 Morrison (Heather's lab) or Testing Room**

1. The ObserverXT license thumb drive (USB) is crucial to opening and using the Observer program on the coding computer in 208 Morrison or on the Playroom computer at AARP. As of January 2021, the thumb drive is permanently plugged into the coding computer in **208 Morrison**. This requires the coder to physically code in Morrison or remote access via TeamViewer to code remotely. Before you try to code remotely open TeamViewer on the coding computer and write down the access code (**1 932 401 472**) and the password (program changes daily?). You will need that code so your remote computer can "talk" to the coding computer. The software will not open without the thumb drive plugged into the computer, do not remove the thumb drive without letting others know!
2. Double-click on the ObserverXT 14 icon (green with white eye icon). In the pop-up window, open the 'CognitiveTestingVaikaDogs…V8 (2)-sub' project file.
3. On Google drive, open the 'Vaika Behavior Coding Log Checklist' (https://docs.google.com/spreadsheets/d/1s3JLCT1EWr3g5h9szsQmIjHfm_rvb6tAD-wJtpgHgvc/edit#gid=0) sheet and first become familiar with the layout. Fill in your name and the test you are coding in the proper cell (i.e if you are coding the 18 month NPT videos scroll to the 18 month timepoint NPT row and write in your name for the NPT column). As a rule, each video needs to be coded by 2 different people in order to assess continuity so there needs to be two coders per test. For example, if I am coding the 18 month OFT, Jean will also code all the OFT videos with me so that we may use both our completed code for analysis. Use the "Dog Age + Sex" tab to find the age of the dog you are coding along with reproductive status (all females have been spayed and some males are neutered). SIDE COMMENT: it is **crucial** to monitor the VetCare channel on Slack for updates on males getting neutered. If a vet neuters a male, you must note the date of neuter in the "Dog Age + Sex" tab to keep track of whether or not a male was intact or neutered during a behavior testing timepoint (as that undoubtedly affects behavior). For example, Lander was neutered 8/27/20 so he was intact for Baseline/PreDrug- 9 month testing but was neutered for his 18-month testing timepoint (and will continue to be neutered for future testing).
4. If using a personal computer or the office computer, the coder will need to download videos from Cornell Box (https://cornell.app.box.com/folder/0) in the various behavior video folders (floater folders not within the large "Vaika Project Cornell" folder).

**2. General Coding**

1. In the ObserverXT program, in the pop-up window, click on the 'Observe' tab. In the new window, click on 'Create a New Observation'.
2. Label the observation with dog's name, test type (OFT, NOT, NPT or MT), testing timepoint and coder's intials - (ex: Trapper_OFT_18months_SC).
3. Select & download the appropriate video file from the timepoint you are coding from BOX, i.e '18 Months Treatment (10.20 Behavior Videos)' folder. When in the Observer program, choose that video to code. New windows should appear - one with a black video box, and one with a list of greyed-out behaviors (Door Zone & Silent).
4. PRO TIP: I find it easiest to make two folders and add it to my "quick access" list within the folder icon on the desktop (bottom of the screen, looks like a manila folder with a blue holder). Name one "completed behavior videos" (with sub folders for timepoints, and sub-sub folders for the dogs in each specific timepoint as you go) and the other "videos to be coded" with raw behavior videos loaded on to be coded. This helps keep things more organized. Make sure to update the 'Vaika Behavior Coding Log Checklist' after every video you code and maintain it regularly to keep information concise for all coders (https://docs.google.com/spreadsheets/d/1s3JLCT1EWr3g5h9szsQmIjHfm_rvb6tAD-wJtpgHgvc/edit#gid=0).
5. Move the video window to the second monitor and enlarge to fill the screen. Expand the first behavior group list (Location) on the right side of the screen. All other behavior groups can remain collapsed.
6. Click the red button (hovering the mouse over the button should show "Start Observation") that is above the grey list. A new pop-up window will appear with several drop-down boxes.
    a. Under 'test', select the appropriate test (for OFT, select OFT; for NOT, select NOT, etc.).

b. Under 'Dog Name', type in the dog's name.
c. Under 'Sex', select the appropriate category for the dog in the video.
d. Under 'Testing Period', select the appropriate period (PreDrug-9 months-18 months etc.).
e. Under 'Age', type in the dog's numeric age in years.
f. Click "Ok".

7. The red button icon will change to a grey square with a red outline - you are now able to start coding the video. **For coding details, scroll down to "Coding Individual Behaviors within Behavior Groups sections.**
8. If the video does not automatically start playing, press the forward play button and use the behavior list on the right-hand side to code the video for the Location behavior group. Recommended speed setting is 1/5x for Location group.
   a. To adjust the time of coded behaviors:
      i. Right-click on the time and select 'Update Time'.
      ii. Change the time by either typing or using the up/down arrows.
9. If the video finishes playing before you have completed all coding, the observation will stop and you cannot code any behaviors. You will need to drag the grey playback slider to the beginning of the video and then click the red button.
10. Continue coding for the remaining Behavior Groups as appropriate for the test (See lists below).
11. Ensure all coded behaviors with modifiers have been set. Click on the modifier; a new pop-up list will appear on the right side of the window from which you can select the appropriate modifier for the behavior. **The modifier must be updated before the next step or error messages will appear and coding cannot be completed**.
12. Once finished, click on the red button to End Observation. A new window will pop-up asking if you want to end the observation - Click "Yes". In the tool bar, click on 'File', then select "Save Project".
    a. If you have not completely coded all behaviors in the video, right click on the observation name in the right-hand menu and select "Rename". Add "partial" to the name (ex: Trapper_OFT_18months_SC_partial). When you have finished coding all behaviors, remove "partial" from the name.
13. On Google drive, open the 'Vaika Behavior Coding Log Checklist' (https://docs.google.com/spreadsheets/d/1s3JLCT1EWr3g5h9szsQmIjHfm_rvb6tAD-wJtpgHgvc/edit#gid=0) sheet and scroll to the timepoint you are coding. Enter "done" in the appropriate column for the dog and test you just finished coding once you have uploaded your ODX, XLS and JPEG completed (exported *PLEASE SEE BELOW*) files onto Box in the appropriate folders.
    a. If coding another video, then click on "Observe", select "Observation", then select "New" and repeat steps 2-12.
    b. If done for the day and coding on the Office Desktop or a personal computer:
       i. Click on "File", select "Export", then select "Observational Data". By default, the program checks all observational data to be exported – **<u>Click "Deselect All" boxes for videos that have already been exported in a previous session and click the one you have just coded</u>**. You will save and export 3 files (The Observer XT, Microsoft Excel and Visualize Data) Check "Include media files" and "Create separate file per observation" under Export Options. Click OK.
       ii. Name the file again with dog's name, test type (OFT, NOT, NPT or MT), testing timepoint and coder's intials - (ex: Trapper_OFT_18months_SC). The files will be saved as .odx and .mp4, **we are only interested in the .odx file in this case.**
       iii. After you successfully exported the .odx file, repeat steps b.1-b.2 but with the **excel observational data.** Be sure to click **"Include User Defined Independent Variables**" at the bottom left of the pop-up screen before you export.
       iv. Once you have exported the .odx and .xls files (and named them properly) click on "visualize data" button at the top of the coding screen to the center-right. This will bring you to a new window with your coded behaviors in graph form. Take a "snip" (like a screenshot on macs) of this and name it as you normally would (ex: Cashew_OFT_18months_SC_pic) and save it with your .odx and .xls files.
       v. Click on "File" and then select "Exit". In the pop-up window, click on "Yes".

vi. Upload the .odx, .xls and .png files into the appropriate folders on box (for example here: https://cornell.app.box.com/folder/124416397508 )
c. If done for the day:
i. Click on “File” and then select “Save Project”.
ii. Click on “File” and then select “Exit”. In the pop-up window, click on “Yes”.

**3. Importing Remote Coding to Testing Room Computer**

1. First, ensure the full license key is plugged into the computer.
2. First, ensure the file has not already been imported by checking the left hand “Observations” group. If not, proceed to step 3.
3. Download the appropriate .ODX and .mp4 files from Cornell Box for the Behavior Testing drive.
4. Click “File”, select “Import”, select “Observational Data”.
5. Ensure .odx is selected in the “File of type” list.
6. Browse to the file and click “Open”.

**4. Initial Coding before 2nd training session with Noldus**

1. Coders will complete all four tests for two dogs: Yale and Trapper.
2. To start, best to code video for one behavior group, then restart video to code next behavior group (replaying the video multiple times) and to start with location group.
    a. Try to keep track of time spent coding videos - will help with scheduling and if behaviors need to be removed for efficiency.
3. Once finished, can decide if
    a. Additional supervision/retraining is required for coders
    b. Coding order will be all dogs for each test, or all tests for one dog.

**5. Coding Individual Behaviors within Behavior Groups**

1. Location - based on front paws/shoulder location: Zones override peripheral. Easiest if coded in slow-motion.
    a. Square Crossed - this is a “count” observation based on when the dog crosses **the black outlined squares** based on the shoulders to help calculate distance traveled. One paw must be touching the ground and the other paw has crossed the line or when the shoulders have passed over the line; general forward motion.
    b. All other behaviors in this group are based on duration. See diagram at end of document (Figure 1) for specifics. Code location changes based on when the shoulders have passed over the boundary lines.
    c. Mobile Divider Zone - Note: this is not outlined in the videos. See diagram (Figure 1) for location.
    d. Mirror Zone is only coded during the Mirror Test. <u>In all other tests the mirror zone is coded as Peripheral.</u>
    e. Other = if dog exits testing space via door or mobile divider

2. Corners - based on front paws/shoulder location. **Must** code start and stop times. Corners are **three grey/pink squares wide**. Code location changes based on when the shoulders have passed over the boundary lines.
    a. Corner A
    b. Corner B
    c. Corner C
    d. Corner D

3. Locomotion - Easiest if coded at normal speed.
    a. Urination & Defecation are count observations.
    b. Walking - based on duration, no modifiers
    c. Trotting - based on duration, no modifiers
    d. Sitting - based on duration, no modifiers - code when the dog is completely sitting.
    e. Standing - based on duration, no modifiers - code when the dog has all four feet on the ground and feet are not moving. Shifting weight or adjustment of foot placement still count as standing.
    f. Laying Down - based on duration, no modifiers - code as dog is laying down.
    g. Bowing - count observations based on when/as occurs. No modifiers.

h. Standing on hind legs only - duration with **modifiers** based on what the dog is putting front paws on (See #12 Stimulus modifier section). Start based on when both front paws have left the ground.
i. Other motion - based on duration - encompasses any locomotion behavior not included in this group (example: leaping or jumping across the room)

4. Circling Locomotion
   a. Circling: based on duration, dog travels in a circular path that is greater in space than one black outlined square. Has **modifier** for direction traveled (clockwise or counter-clockwise)
   b. Tight spinning - walking/trotting in circles in the estimated size of one black outlined square. Based on duration, has the **modifier** for direction traveled (clockwise, or counter-clockwise).

5. Sniffing Interactions
   a. Sniffing - based on counts. Mark the beginning of 'episodes' when dog is sniffing room components/objects. Has **modifier** for stimulus in room. Each time the dog stops sniffing and then resumes should be counted as separate episodes.

6. Vocalizations - best if coded at normal speed to accurately discern dog vs room sounds - use headphones or speaker to increase sound level.
   a. Barking - based on duration of episodes when you hear barking - pauses in episodes can be coded as 'silent'. Has **modifiers** based on type: Aggressive, Playful, or Regular.
   b. Yip - high pitched quick vocalizations; distinctly not a whine and not a bark (Yale OFT for example) coded as a count. (Inactived in coding software).
   c. Whining - based on duration of episodes. No modifiers.
   d. Growling - based on duration of episodes. Has **modifiers** based on what dog is growling at (Stimulus modifiers)
   e. Howling - based on duration of episodes. Has **modifiers** based on what dog is howling at (Stimulus modifiers).

7. Room Interactions - Mutually exclusive, must code start and stop points for durations.
   a. Scratching - count observations based on when you hear contact. If there is an intense scratching episode, only code at the beginning of the episode. Has **modifier** for stimulus in room.
      i. During NOT - do not code for scratching toys.
      ii. During NPT - do not code for scratching person.
      iii. During MT - do not code for scratching mirror.
   b. At door opening side - based on duration that dog spends in the door zone/Corner A with their nose pointing towards the opening side of the door.
   c. At door hinge side - based on duration that dog spends in the door zone/Corner A with their nose pointing towards the hinges of the door.
   d. Hinge side vs opening side:
      i. If dog's body is in the pink square and the dog's head is pointed straight at the door, this counts as "at door opening side".
      ii. If the dog's body is in the grey square and the dog's head is pointing straight at the door, this counts as "at the door hinge side".
      iii. If the dog's body is overlapping the pink and grey square, code based on <u>where the dog's nose is pointing</u>.

8. Toy Interactions - Only coded during NOT
   a. Not interacting with toys - based on duration of dog not interacting or looking at toys
   b. Interacting with toy - based on duration of when dog is interacting with a toy that does not include sniffing the toy (pawing, licking, carrying, nose bump, etc). Has **modifier** for specific toy (Turtle, Kong, or Cone).

9. Human interactions - Only coded during NPT
   a. Not interacting with person - based on duration that the dog is not looking at or interacting with the person

   b. Interacting with person - based on duration that dog is specifically interacting (licking, pawing) with the person on the legs, hands, body, face, or chair.

10. Mirror interactions - Only coded during MT
    a. Not interested in mirror - duration of time that the dog is not interacting with or looking at the mirror.
    b. Interacting with mirror - duration of time dog is directly in front of the mirror looking at their reflection, licking, etc.
    c. Run into mirror - count of dog making contact with the mirror with their head (not just a nose touched; tried to walk/run through mirror).
11. Tail Posture - Tail Wagging modifiers for all
    a. Tail Not tucked/low - duration of time tail is level with back or up over the back. When sitting or laying down, the tail is out behind body.
    b. Tail tucked/low - duration of time tail is hanging down between legs or is tucked under body. Might be hard to see tail from above. When sitting/laying down, tail is beneath body.
    c. Wagging Modifiers:
        i. No wagging - no side-to-side movement.
        ii. Wagging - there is side to side movement.
12. Ear Posture
    a. Ears pinned back - Ears are tucked and flat against the head.
13. General Overall Impression (will be placed in the 'Vaika Behavior Coding Log Checklist' (https://docs.google.com/spreadsheets/d/1s3JLCT1EWr3g5h9szsQmIjHfm_rvb6tAD-wJtpgHgvc/edit#gid=0) code at the start of the video. Select one behavior after watching the entire test; this behavior will be coded for the entire video length.
    a. Curious - willing to approach novel objects/person; investigating room with sniffing, willingness to interact with the environment; not just circling the room
    b. Neutral - might be moving about room, but don't really care about the room (not stressed, slinking, excited, etc.). Dogs may vocalize and scratch at door, but it's not "stressed".
    c. Disoriented - seems uncertain about surroundings, generally confused and lost; doesn't interact with environment as expected
    d. Nervous/Fearful - remains in one place for long periods of time; general body postures are low to the ground, slow slinking movement in room; likely observes from a distance. May approach objects hesitantly. Possible vocalizations of growling and barking.
    e. Happy/Excited - Running around the room; quickly checks objects, but then moves on to something new. Lots of tail wagging, quick movements.
    f. Social - only for NPT & MT - very interested in the person/mirror reflection; continually approach and interact. Lots of tail wagging and licking, and seeking reciprocal attention.
    g. Aloof - only for NPT & MT - general disinterest in the person/mirror reflection. They acknowledge the presence of the person/reflection, but don't interact (not based on fear).
    h. Separation Anxiety - primarily focused attention on door. Might scratch or vocalize at door. May interact with room/objects.
14. Stimulus Modifiers
    a. Floor
    b. Wall - includes white pipes, metal duct, and hose
    c. Metal Grate (full length along the two walls)
    d. Door
    e. Mobile divider
    f. Novel person
    g. Turtle toy
    h. Kong toy
    i. Cone toy
    j. Mirror
    k. Other - anything not included in the list above

**6. Coding Checklist**

6.1. Coding required for OFT

1. Location - do not code Mirror Zone
2. Corners
3. Locomotion
4. Circling Locomotion
5. Sniffing Interactions
6. Vocalizations
7. Room Interactions
8. Tail Posture
9. Ear Posture
10. General Overall Impression.

6.2. Coding required for NOT

1. Location - do not code Mirror Zone
2. Corners
3. Locomotion
4. Circling Locomotion
5. Sniffing Interactions
6. Vocalizations
7. Room Interactions
8. Toy Interactions
9. Tail Posture
10. Ear Posture
11. General Overall Impression - from ZC

6.3. Coding required for NPT

1. Location - do not code Mirror Zone
2. Corners
3. Locomotion
4. Circling Locomotion
5. Sniffing Interactions
6. Vocalizations
7. Room Interactions
8. Human interactions
9. Tail Posture
10. Ear Posture
11. General Overall Impressions - from ZC

6.4. Coding required for MT

1. Location
2. Corners
3. Locomotion
4. Circling Locomotion

General Layout/Location

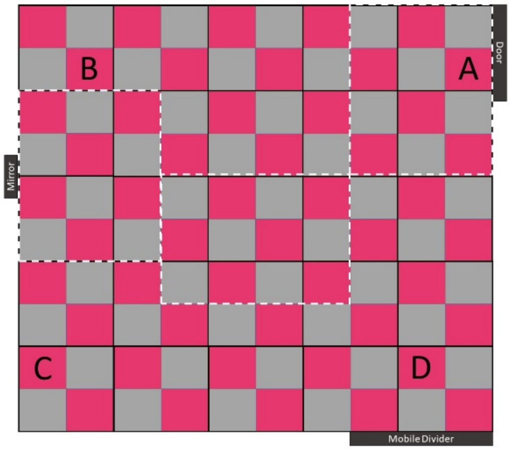


Corners coded separately from Zones; Zones override Peripheral.
Other=exit testing space via door or mobile divider

Zones

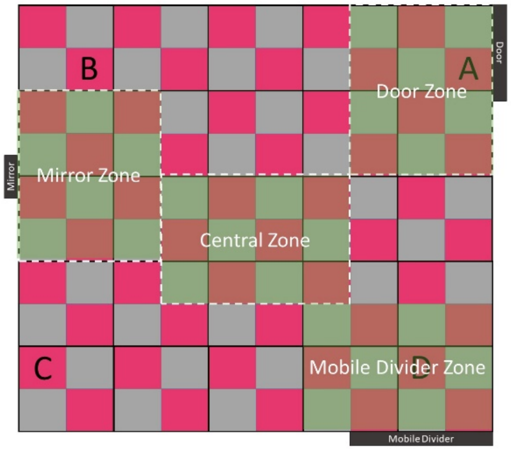


Locations

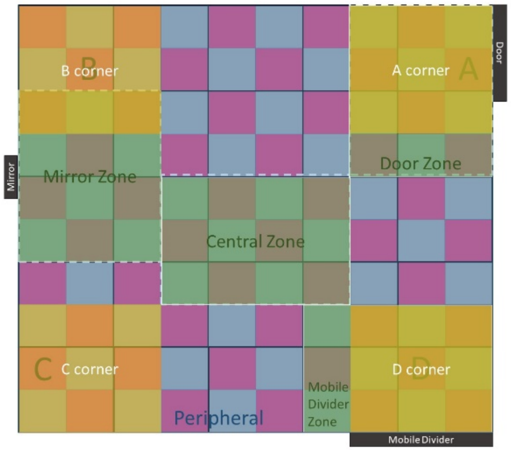


Corners coded separately from Zones; Zones override Peripheral.
Other=exit testing space via door or mobile divider

## V. Detour ("V") Behavior Test (Adapted from Penn Vet Working Dog Center, Dr. Cindy Otto)

**1. Before Testing Begins**

1.1. Open document files

- Google file:
  https://docs.google.com/spreadsheets/d/1deYPafU3PyGUUkXltxalgG46PtYaLD6fC6NEU4dUsrk/edit?usp=sharing
  - File has 2 tabs per testing. "Test_1_Oct2020" is the list of dogs (vertically) tested with trial variables and times. "Behavior_Test_1" is the list of dogs (horizontally) with each behavior attribute and score.
- Box file:
  https://cornell.app.box.com/folder/124752427257

1.2. Gather required supplies

- Stopwatch or phone w/ stopwatch function
- Paper data records (blank forms) and clipboard
- Retrieval / Motivation item (**only 1 item used per dog; must be consistent across time points to use the same item for specific dog every time**)
  - Food, treats, and dog bowl as needed with a complementary set for outside the playroom in the waiting area to reward dogs between trials (canned cat food, cheese wiz); extra food should not be in the testing area during trial.
  - Toy (tug, kong)
- See-through metal fencing panels that are self-standing and can be put in a V-shape
- Four cinder blocks (used to stabilize fencing in upright position)
- Camera: Noldus camera to capture behavior videos.
- People: Minimally 3 people, a 4th person is helpful.
  - Experimenter: times trials and scores behavior – should remain consistent person through entire test for all dogs and be a person the dogs are familiar with as person will be in testing area with the dogs; may need to do assistant duties as well but MUST wash hands thoroughly before dog comes in to test.
  - Handler: handles the dogs during each test; should remain consistent or limited to 2 people throughout entire test; should be a person the dogs are very familiar with.
  - Camera Operator (and data entry): operates Noldus camera and enters data from hard copy to google sheet; person sits behind the portable dividers at computer.
  - Assistant: gets food /treat prepared and in bowl and alerts handler when it is time to bring the dog into the room; goes outside into the hallway during the actual test.

1.3. Setup

- Test will occur in the Playroom at AARP facilities within the behavior testing area (floor covered by squares) with the divider separating the behavior area from the treadmill area.
- A Noldus camera is used to capture behavior videos. Start recording when dog is brought into room to see food/toy. Maintain single recording throughout all tests for an individual dog. The following steps are followed by the Camera Operator to capture and save videos:
  - Media Recorder 4 (Red icon on desktop)
- On the desktop, double-click the Media Recorder icon (red with white camera icon) to turn on the camera. Once the program opens, select "File", then "Preferences", then "N-link settings", then "Test Connection". Make sure the window reads "Connected". Click "Ok".
- In the Media Recorder program, select "File", then "Video Settings…". In the pop-up window, ensure the check box is filled next to "Use maximum recording time". Change the time to "00:15:00" to record for 15 minutes. Click "Apply".
- Make sure the video is set to "RTSP IP Camera (Noldus RTSP Source F…)" and the audio is set to "Jack Mic (Realtek® Audio)". This should be automatically set but if it's not, go to "Settings" and click on "Video device" and "Audio device" and select the correct devices. It should look like this:

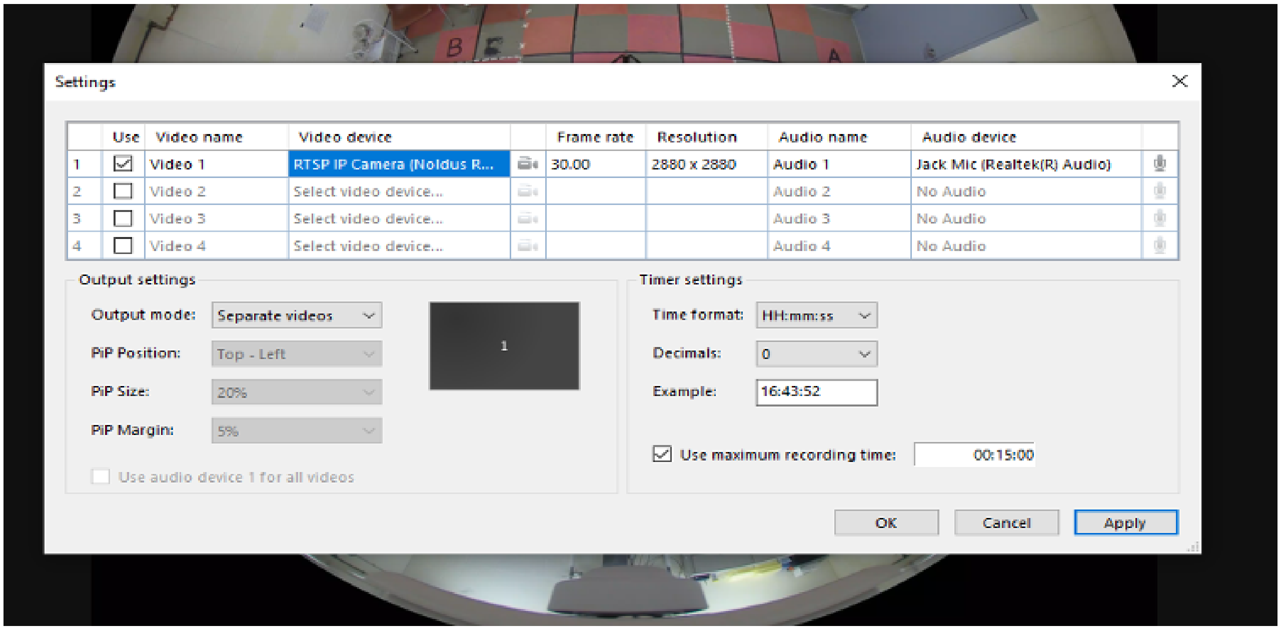


- After you have set the correct audio + video devices and timer, it is time to make a folder for the videos to save to. If doing multiple days of testing it is easiest to create a master folder with sub folders for each day of testing. Make a folder called "V Detour Testing (date)" in your File Explorer with sub folders for each day you test. Go to the bottom right of the media recorder interface and click the box with three dots:

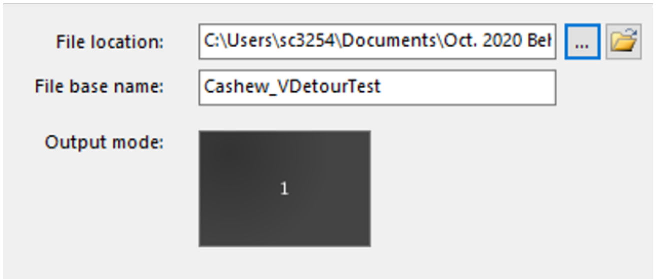


- Once clicked a pop up window will appear for you to select the folder you want the videos to save to. Find the master folder you made then click to find the sub folder with the day of testing. Once found, select it and click "ok".
- You can check to see if you chose the correct folder by clicking the little folder opening icon next to the icon with the three dots. This is "Open Target Folder" and is useful should you need to open that folder to check something.

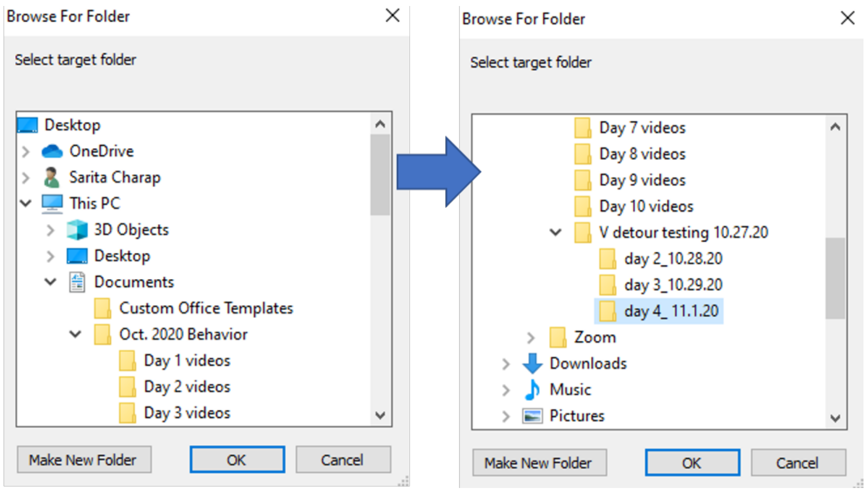


- For the "File base name" window below the "File location" window you should be as descriptive and consistent for each dog you record. For example, I have chosen Cashew_VDetourTest for the first detour test Cashew the dog has done. When Detour Testing happens again Cashew's tests will be Cashew_VDetourTest_6month or something along those lines. Keep that format for each dog (i.e Tomcat's file will be Tomcat_ VDetourTest and in 6 months will be Tomcat_ VDetourTest_6month).
- **PRO TIP**: Occasionally click the "Refresh" button next to the "Settings" button to cut down on the number of times the video freezes. If the video freezes you will have to click the red "Record" button to stop recording (even though it's frozen) and begin the trial over again (it's a pain).

- After the day of testing, you will upload all videos to BOX in the V Detour Test folder that is outside the Vaika Project Cornell folder and next to the Cognitive Dysfunction Behavior video folders (https://cornell.app.box.com/folder/124752427257). Make a separate folder within the V Detour Test folder for the specific time point you tested on.

- Fence will be put in a V-shape with motivation object placed inside/outside of the apex of the V and dog started at the opposing outside/inside area. The fence is 2.4 meters in length on each arm and set at an angle of 80 degrees. Fence is metal and see-through. The experimenter is seated in the upper left-hand corner of the room as viewed from above (corner "B"), opposite the door.

- Three trials will be conducted with dog/handler on the outside of the apex of the V (Figure 2A). The 4th trial will have the handler and dog on the inside of the apex of the V (Figure 2B). Note that experimenter stays in the same corner (corner "B") for all trials for Vaika tests.

**2. Test Start**

1. Record dog name, AM playtime, feeding time, test date, test time, target (food/toy), handler, and experimenter for the dog.
2. Camera should be set to record and continue through all 4 repetitions.
3. Toy will be played with each dog and its handler before each trial for a maximum of 15 seconds or small amount of food will be given to each dog before each trial.
4. Dog should then be taken by their handler outside to relieve themselves for ~3-5 minutes or until they pee/poop, out of sight of the V-shaped fence.
5. When the dog is out of sight, the toy or food bowl ("motivational item") should be placed behind the V-shaped fence by the experimenter. Taking the dog out of the room is a precautionary step intended to prevent the dog from learning the detour task as experimenters place the toy/bowl in the appropriate position.
6. Once the toy/food is placed, the handler should bring the dog into the room and to the starting point.
    a. Starting point is directly in front of the fence, on the opposite side of the reward, in front of the reward.
    b. Handler should make sure the dog is settled and focusing on the apparatus as much as possible before the trial begins.
7. Dog is released by the handler at the signal of experimenter who starts the timer. The dog is given 30 seconds to navigate the V-shape obstacle.
    a. The trial begins once the leash/collar is released.
    b. A behavior score for the 6 characteristics described below (Section 3) should be recorded per trial.
    c. The trial will be done with the "inward V" setup (Figure 2A) for 3 consecutive repetitions with only the time to reset the equipment, timer, and move the dog in between each repetition.

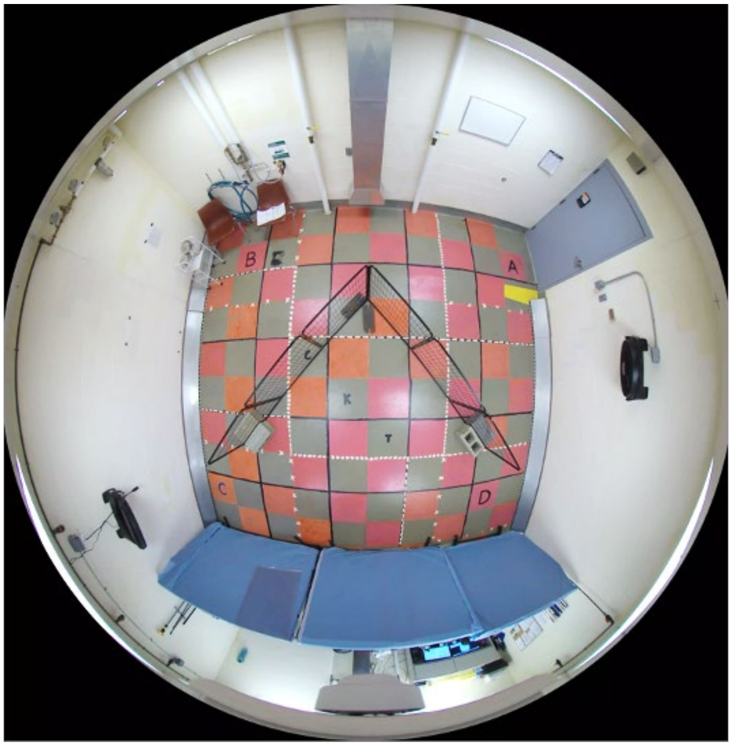


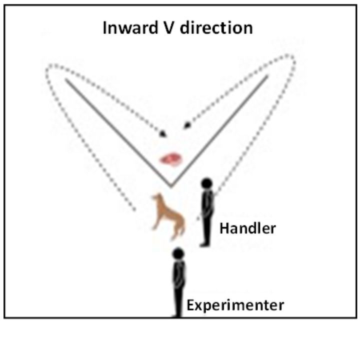


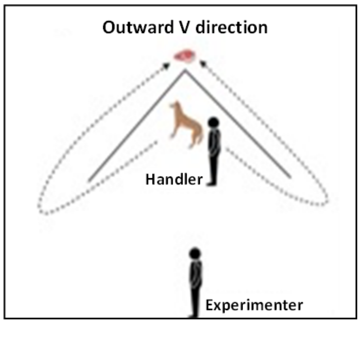

8. The dog will be completing a detour task, where they are expected to detour around a V-shaped fence to retrieve a toy or food. **The success or failure and time of successful trial completion will be recorded for each individual trial.** The direction (**left or right from the perspective of the seated/consistently stationary Experimenter**) that the dog goes around obstacle should be recorded when appropriate.
9. After a successful trial, the handler will then play with the dog (maximum 15 seconds) or give the dog the small amount of food in the bowl and take the dog out of the room. Should a dog fail to navigate the detour to the food/toy, the dog will be leashed with minimal handler interaction (no reward) and taken out of the room.
10. Between trials, the dog should then be taken out of the room and out of sight of the V-shaped fence by the handler while the toy/food is being placed again. Handler and dog will be in waiting area where the same food/treats/toy are available and the dog should be rewarded briefly while room is prepared again. Should a dog fail to retrieve target object, the dog will be rewarded to a lesser extent outside the playroom.
    a. Dogs should <u>not</u> see the target item being retrieved at any time by handlers or experimenters. This is another precautionary step to avoid dogs learning paths to the target.
11. After three trials, the experimenters will reverse the set-up of the see-through fence to create an "inward V" (Figure 2B). This changes the path required for the dog to navigate to the target object and tests the dog's ability to problem solve in a different scenario. One trial is performed with the "inward V" setup.
12. Once all 4 trials are completed, the dog may be returned to its kennel.
13. After a dog has completed their 4 trials, the room will be mopped for urine (with 1:64 Rescue or Perioxigard) and prepared for the next dog. Do not worry about mopping urine between trials (a dog may be in the presence of their own urine but **not** a different dog's urine as it will be distracting). Please remove feces immediately between trials should a dog poop.
14. Behavior parameters (see below, Section 3) should be scored as an overall assessment of the dog's performance throughout all 4 trials as well.

**<u>3. Behavior parameters to score during testing</u>**

Each dog is given behavior performance ratings for the following parameters after each trial and an overall rating for their test (all four trials combined). Score range of 1-3 is used for each parameter as described below.

A. *<u>Distractibility</u>*- how focused the dog is on the detour task
    a. 3 Extremely distracted (not focused on detour task at all, sniffing, walking around but not trying to get to target)
    b. 2 Somewhat distracted (tries to get to target but easily distracted and does other activities)
    c. 1 Not distracted (focuses on target entire time)
B. *<u>Nervousness</u>*- tendency to demonstrate behaviors associated with nervousness or anxiety
    a. 3 Very nervous (tail tucked, ears down, potentially shaking)
    b. 2 Somewhat nervous (occasionally demonstrates nervous/anxious behavior)
    c. 1 Not nervous (does not appear nervous or anxious, in contrast, dog may seem excited or happy – tail wagging, head up and ears perked)
C. *<u>Handler interaction</u>* – how much the dog interacts or focuses on the handler
    a. 3 Handler focused (totally focused on handler and disinterested in target)
    b. 2 Handler mild interest (occasionally looks or goes to handler but also looks at target)
    c. 1 Handler disinterest (not interested or interactive with handler)
D. *<u>Experimenter interaction</u>* – how much the dog interacts or focuses on the experimenter
    a. 3 Handler focused (totally focused on handler and disinterested in target)
    b. 2 Handler mild interest (occasionally looks or goes to handler but also looks at target)
    c. 1 Handler disinterest (not interested or interactive with handler)
E. *<u>Target motivation</u>* – how much the dog is motivated by the target object outside of the trial scenario
    a. 3 Not Target Focused (not interested or motivated by the target object in any situation)
    b. 2 Moderate Target Focus (occasionally interested and motivated by the target object outside of the trial situation, may paw or push on the fence once or twice to try and get to target)
    c. 1 Extremely Target Focused (very focused and interested in the target object in nearly any situation, may paw or push on the fence repeatedly, ie. very food or toy motivated)

F. *Spatial disorientation* – assessment of the dog's "accidental" interaction with the V-shape fence itself. Dogs demonstrating "Awkward (3) or Moderate (2)" scores should be assessed for vision problems and/or neurological ataxia within 2 weeks of testing date as these both can be related to clumsiness and bumping into things.

a. 3 Awkward (commonly bumps into the fence accidently (≥3 times), general dog locomotion is clumsy or dog seems disoriented; does NOT include basic stiff or lame gait as long as the dog is moving in a coordinated fashion)
b. 2 Moderate (occasionally bumps into the fence inadvertently (1-2 times) but generally walks around ok)
c. 1 Fine (dog locomotion is controlled and not demonstrating any clumsiness, dog may have a stiff or lame gait but is not disoriented when walking).

## VI. Necropsy Tissue Sample Collection Protocol

In the event that a dog must be humanely euthanized or passes naturally, a variety of tissues samples will need to be collected.

**1. Before euthanasia and/or necropsy**

Due to complexity and logistics of necropsy, when able, all dogs should be taken to the Diagnostic Laboratory and an AHDC Necropsy form should be used (see attached). If a dog is found deceased in a kennel or passes during the day, a necropsy should be performed as soon as possible. Contacting Dr. Loftus, Dr. Huson and Dr. Wakshlag should be attempted and the first one found will help facilitate the necropsy with the Diagnostic Laboratory personnel. The pathologist Dr. Andrew Miller will be notified by one of the Doctors – but there are necropsy technicians that should be notified as they will help facilitate the procedures.

Drs. Wakshlag, Huson or Loftus will contact any other research parties that have an MTA agreement in place to collect certain tissues during the procedure. Vaika will receive priority for sample collections and other parties will be allowed to participate after all collections have been done.

At the determined time for euthanasia, the Vaika LVT will transport the dog to the ECRF. Location will be dependent on the type of euthanasia, which depends on who has expressed interest in sample collection.

If the dog is to be euthanized on site at the Diagnostic Laboratory, please also contact Dr. Loftus and Seth Peng as dogs will be having blood collected and a sterile skin sample will be collected to propagate fibroblasts in cell culture.

The Vaika LVT will be required to bring all necessary supplies for sample collection as the ECRF Necropsy room is not stocked with these supplies for Vaika use. A prepared Necropsy Kit can be found in the top drawer of the file cabinet in the AARP office. This kit along with the dog's binder should be brought to the ECRF. This kit contains:

- Cardstock and Black Ink Pad (for paw prints)
- Medium Nitrile Gloves
- Large Weigh Boats
- #10 Scalpel Blades
- Micro tubes and Tube Rack
- Markers and Pens
- Notepad
- Plastic Sandwich bags
- Blue Surgical Clothes
- Small Metal Bowl
- Box Tape
- LN2 Handling Gloves

Some additional supplies will need to be added to this prior to sample collection. These items include:

- Scientific Label Maker (found in the top drawer of the Vaika cabinet)
- Clippers (found next to the printer on top of the Vaika cabinet)
- Surgical Instruments (found in the red cold sterile tray in the top drawer of the Vaika cabinet, these items should be removed from the cold sterile and place into the Necropsy Kit for travel)
    - Scalpel Handle
    - Tissue Forceps x2
    - Surgical Scissors
    - Haemostats
- Stethoscope (found hanging on the partition in the playroom)
- Sample storage box (found in top drawer of the Vaika cabinet)
- Slotted spoon (found in the bottom drawer of the Vaika cabinet)
- PBS wash bottle should be filled (additional PBS can be found in the top of the Vaika storage cabinet in the storage room)

If possible, micro tubes should be prepared and labelled prior to travel to the ECRF. Tubes should be labelled with the label maker. Labels should include the following information:

- Dog's name and ID#
- Date
- Sample Type
- Sample Number (A,B,C, etc.)

The following samples should be collected as approximately 500mg tissue samples (about the size of 3 peas; however, some tissues such as the lungs require a larger sample size due to their light weight):

**Tissue Trim List for Sled Dog Study**

All organs to be collected in 10% formalin as well as collection for snap freezing of tissue):

1. Spleen (3 frozen samples)
2. Popliteal lymph node
3. Thyroid
4. Adrenal
5. Pituitary
6. Esophagus
7. Trachea
8. Gall bladder
9. Right medial liver lobe (3 frozen samples)
10. Right lateral liver lobe
11. Heart (LV) (2 frozen samples)
12. Lung (left cranial and right cranial) (3 frozen samples)
13. Stomach (fundus and pylorus)
14. Pancreas (left lobe)
15. Duodenum
16. mid-jejunum
17. ileum
18. Cecum
19. Colon
20. Mesenteric LN
21. Kidney (transverse mid-section through left kidney – 3 frozen samples)
22. Bladder
23. Reproductive organs based on sex (prostate & testes – from males that are intact; mammary – from females)
24. Muscle (gastrocnemius, triceps, biceps femoris – 2 frozen sections biceps femoris and one for frozen sectioning – isobutanol collection when convenient)
25. Skin – neck region
26. Aorta proximal to heart
27. Eye (no frozen sections)
28. Falciform fat and popliteal fat (2 sections frozen of each)
29. Bone Marrow (no frozen sections)
30. Synovium from Stifle
31. Tibial cross section – mid (no frozen sections)
32. Brain sections, left and right, for:
    - Frontal cortex
    - Parietal cortex
    - Occipital cortex
    - Caudate nucleus (no frozen sections)
    - Hippocampus
    - Cerebellum (left and right)
    - Thalamus (no frozen sections)
    - Pons (no frozen sections)
    - Medulla (no frozen sections)

33. Spinal cord: Take entire spinal cord at necropsy and trim C1, T1, L1 from all animals (by counting nerve roots – (1 frozen section from L1)
34. Sciatic nerve

Any apparent solid tumors should also be collected for histopathology and frozen storage (3 samples frozen)

Before leaving for the euthanasia location, the cryopod will need to be filled with liquid nitrogen (LN2). The cryopod can be found in the bottom drawer of the Vaika file cabinet. To fill the cryopod, please see the LN2 Collection Protocol.

**2. Performing necropsy**
On arrival, the CARE veterinarian performing the euthanasia (Dr. Loftus or Dr. Wakshlag) should be notified of your arrival and will aid in bringing the dog inside the secure building.

To enter the Show and Tell Necropsy room, shoe covers and coveralls or scrubs will need to be worn. Only necessary items are to be brought into the room. During the winter, coatsshould be left in the locker room. Before commencing with the necropsy, set up supplies on the work space next to the show and tell necropsy. The pathology technician and/or resident will be helping to collect tissues for formalin fixation while tissues for freezing will be collected on the other side of the table.

Prior to the start of the necropsy, ink prints of the paws should be collected to keep at AARP. Paws with long nails or fur between the toes should be trimmed back using nail trimmers and electric clippers. The paw should be pressed into the black ink pad until saturated, then pressed firmly onto the card stock. Additional ink pad refill is available if needed.

Collect all tissue samples indicated above in the Tissue Trim List for Sled Dog Study. Also, as noted above, collect any skin masses or tumors for both histopathology and frozen storage.

If completing the frozen tissue collection without assistance:
- Check the dog's binder to confirm the location of any known masses or tumors (see body map).
  - These areas should be shaved once the mass has been located to facilitate collection. Organ tissues should be collected first (before masses/tumors) to reduce tissue degradation.
- Samples do not need to be collected in a specific order; however, organ samples should be collected before searching for lymph nodes, mammary samples or dermal samples.
- It is not always possible to locate the prostate on a neutered male dog. If unable to find the prostate and retrieve a sample, this is alright. If there is a known prostate abnormality and you are having difficulty locating it, seek the help of the necropsy technician or pathologist.
- Place each sample into its own micro tube. Snap freeze the sample as soon as possible by dropping the tube in the LN2-filled cryopod. To prevent LN2 from evaporating, keep the lid of the cryopod on as much as possible. Several samples may be dissected and placed in tubes before placing them all into the LN2 to reduce how often the lid is opened. The samples can remain in the pod until ready to place in the -80°F freezer.

**3. After necropsy**
Once collections have been completed, surgical instruments should be cleaned at one of the sinks as best as possible; they can be cleaned more thoroughly upon return to AARP. Clippers should also be cleaned as best as possible and thoroughly cleaned once back at AARP. Repack the Necropsy Kit. Check to ensure all supplies have been cleaned and collected.

Frozen samples will need to be taken to the -80°F freezer located adjacent to Dr. Loftus' and Wakshalg's labs. Once at the freezer, use the slotted spoon to remove the samples tubes from the LN2 and place into a storage box as outlined by the Vaika Sample Storage Protocol. Gloves should be worn when handling the cold samples to prevent skin burns. Label the box using pink tape and a label from the label maker stating the appropriate information as outlined in the Vaika Sample Storage Protocol. Once samples are placed in the box and labelled, place the box in the appropriate rack in the freezer.

When back at AARP, return the Necropsy Kit to the top drawer. Dirty clothes should be washed in the laundry. Surgical instruments should be thoroughly cleaned at the sink and placed back in their cold sterile tray with fresh solution (can be found in the top of the Vaika storage cabinet). Clippers should be thoroughly cleaned using the clipper brush, clippercide, and if needed, blade wash. Return the clippers to their charging station on top of the Vaika cabinet. The dog's collar and paw print can be hung in the AARP office along with the others.
